# Investigation of the potential effects of estrogen receptor modulators on immune checkpoint molecules

**DOI:** 10.1038/s41598-024-51804-2

**Published:** 2024-02-06

**Authors:** Nikita Abramenko, Fréderic Vellieux, Kateřina Veselá, Zdeněk Kejík, Jan Hajduch, Michal Masařík, Petr Babula, David Hoskovec, Karel Pacák, Pavel Martásek, Karel Smetana, Milan Jakubek

**Affiliations:** 1 BIOCEV, First Faculty of Medicine, Charles University, 252 50 Vestec, Czech Republic; 2 Department of Paediatrics and Inherited Metabolic Disorders, First Faculty of Medicine, Charles University and General University Hospital, 120 00 Prague, Czech Republic; 3 Institute of Anatomy, First Faculty of Medicine, Charles University, 120 00 Prague, Czech Republic; 4 Department of Physiology, Faculty of Medicine, Masaryk University, Kamenice 5, 625 00 Brno, Czech Republic; 5 Department of Pathological Physiology, Faculty of Medicine, Masaryk University, Kamenice 5, 625 00 Brno, Czech Republic; 6 1st Department of Surgery-Department of Abdominal, Thoracic Surgery and Traumatology, First Faculty of Medicine, Charles University and General University Hospital, U Nemocnice 2, 121 08 Prague, Czech Republic; 7 Section on Medical Neuroendocrinology, Eunice Kennedy Shriver National Institute of Child Health and Human Development, National Institutes of Health, Building 10, Room 1-3140, 10 Center Drive, Bethesda, MD 20892 USA

**Keywords:** Biological techniques, Biotechnology, Cancer, Computational biology and bioinformatics, Immunology

## Abstract

Immune checkpoints regulate the immune system response. Recent studies suggest that flavonoids, known as phytoestrogens, may inhibit the PD-1/PD-L1 axis. We explored the potential of estrogens and 17 Selective Estrogen Receptor Modulators (SERMs) as inhibiting ligands for immune checkpoint proteins (CTLA-4, PD-L1, PD-1, and CD80). Our docking studies revealed strong binding energy values for quinestrol, quercetin, and bazedoxifene, indicating their potential to inhibit PD-1 and CTLA-4. Quercetin and bazedoxifene, known to modulate EGFR and IL-6R alongside estrogen receptors, can influence the immune checkpoint functionality. We discuss the impact of SERMs on PD-1 and CTLA-4, suggesting that these SERMs could have therapeutic effects through immune checkpoint inhibition. This study highlights the potential of SERMs as inhibitory ligands for immune checkpoint proteins, emphasizing the importance of considering PD-1 and CTLA-4 inhibition when evaluating SERMs as therapeutic agents. Our findings open new avenues for cancer immunotherapy by exploring the interaction between various SERMs and immune checkpoint pathways.

## Introduction

Immune checkpoints play key roles in regulating the immune system response. The design and development of new inhibitors or repurposing clinically used drugs are important areas of research. Several high-impact studies have shown promising results, suggesting that flavonoids, also known as phytoestrogens, could be potent inhibitors of the PD-1/PD-L1 signaling axis. Therefore, we investigated the potential use of estrogens and estrogen receptor modulators as inhibiting ligands of PD-1, PD-L1, and CTLA-4 using molecular docking methods. The calculated binding energy values indicate that quinestrol, quercetin and bazedoxifene could potentially exhibit therapeutic effects through the inhibition of PD-1 and CTLA-4.

Estrogens are among the most important hormones that control not only reproduction in females, but also play a significant role in the overall regulation of the female organism throughout the fertile period. This was highlighted during the COVID-19 pandemic, where the ratio of infected women to men was similar, but the mortality rate among males was higher in countries with limited access to medical care or lower levels of health care resources^1^. In addition to their apparent anti-viral effects, estrogen factors have been shown to support the function of cardiovascular systems^2^ and respiratory systems^3^. These molecules also help prevent endothelial damage^2^ and minimize the risk of cytokine storm^4^ by reducing the binding of IL-6 to its receptor^5^. Estrogens, including the phytoestrogens found in the diet, have been found to influence wound healing, cancer microenvironment, and viral infections such as COVID-19^1,6,7^. These processes intersect at an important crossing point: the microenvironment changes represented by IL-6-dependent inflammation^8^. These findings have sparked interest in exploring the role of estrogen receptor modulators in the immune system, particularly in relation to immune checkpoints, and their potential clinical applications.

The immune system serves as an effective protective mechanism against various pathogens, including tumor cells. In the context of anti-tumor immune response, the activation of T cells plays a key role, which requires fulfilment of two conditions^9^: first, an antigen-presenting cell (APC) must present antigens to T cells through the interaction between the peptide-presenting major histocompatibility complex (MHC) molecule and the T-cell receptor; second, co-stimulatory molecules must be activated. Without proper co-stimulation, T cells enter a state of clonal anergy in which they become unresponsive. Tumors often evade immune surveillance by downregulating both MHC and co-stimulatory molecules while upregulating co-inhibitory molecules. Two representative immune checkpoint proteins are programmed cell death 1 (PD-1) and T-lymphocyte-associated antigen 4 (CTLA-4)^10^. PD-1 shares 21–31% sequence identity with CTLA-4^11^, but unlike PD-1, CTLA-4 contains an extracellular cysteine residue that allows it to form covalently bound homodimers.

PD-1, also known as CD279, is a surface transmembrane glycoprotein and a member of the CD28 family^12^. It is not expressed on resting naïve T cells but is found on the surface of TCR-stimulated T cells^13^. PD-1 has two known physiological ligands: programmed death-ligand 1 (PD-L1; B7-H1; CD274) and programmed death-ligand 2 (PD-L2; B7-DC; CD273)^14^. PD-L1 can be expressed by T and B cells, dendritic cells (DCs), macrophages, and cancer cells, while PD-L2 is present on cancer cells, macrophages, dendritic cells, and B cells. PD-1 represses the immune response by suppressing the activity of T cells and protects the body against chronic inflammation. Nevertheless, in the tumor environment, PD-1 expression induces an immunosuppressive phenotype. The interaction of PD-1 with PD-L1 activates the Src homology region 2 domain-containing phosphatase-1 (SHP-1) and, to a higher extent, SHP-2^15^. SHP-1 and SHP-2 suppress T-cell receptor (TCR) function, leading to inhibited cell proliferation and cytokine production, such as that of interferon-γ (IFN-γ) and interleukin 2 (IL-2)^13^. However, in regulatory T (TREG) cells, the PD-1/PD-L1 signaling axis stimulates cell proliferation and Foxpro3 signaling^14^. Furthermore, IFN-γ produced by activated NK and T cells can induce PD-L1 expression in cancer cells^16^.

In contrast to PD-1, CTLA-4 exerts its immunosuppressive effects on T cells during the early phase of immune response. The activation of T cells involves the interaction between antigen/MHC and the T-cell receptor (TCR), or between CD80/86 on antigen-presenting cells and CD28 on T cells^17^. However, the presence of CTLA-4 suppresses the activation of T cells. CTLA-4 interacts with CD80/CD86 on the surface of antigen-presenting cells, including dendritic cells and macrophages^10^. Additionally, CTLA-4 expressed by TREGs can stimulate trans-endocytosis of CD80/CD86 in dendritic cells, thereby suppressing their activation function^17^.

Numerous inhibitors targeting the PD-1/PD-L1 and CTLA-4 signaling pathways have been discovered^18–20^. However, the development of novel inhibitors or repurposing existing compounds remains of great importance. Flavonoids, also known as phytoestrogens, have been reported to exhibit potent inhibitory activity against the PD-1/PD-L1 signaling axis^21–23^. Similarly, other ligands of estrogen receptors, such as selective estrogen receptor modulators (SERMs), have the potential to act as inhibitors of immune checkpoint proteins. In the process of identifying protein target ligands, molecular docking is a valuable computational tool employed for studying the interaction of a set of SERM molecules with immune checkpoints.

Considering the chemical similarity between quercetin and certain SERMs (e.g., Tanimoto similarity indices between quercetin and luteolin or genistein are 0.7927 and 0.5536, respectively), additional ligands of estrogen receptors like SERMs could serve as inhibitors of immune checkpoint proteins^24^. To address the need for introducing novel molecules and repurposing existing compounds to target immune checkpoint proteins, we conducted a computational analysis utilizing molecular docking. Specifically, we examined the possible interactions between a set of 14 SERM molecules and three immune checkpoint proteins, namely CTLA-4, PD-1, and PD-L1, along with one of the physiological ligands of CTLA-4, the CD80 protein. Quercetin was used as a reference compound in this in silico study.

**Figure 1:**
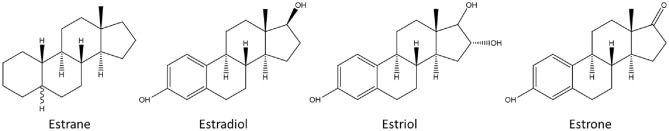
Chemical structures of estrogens.

**Figure 2:**
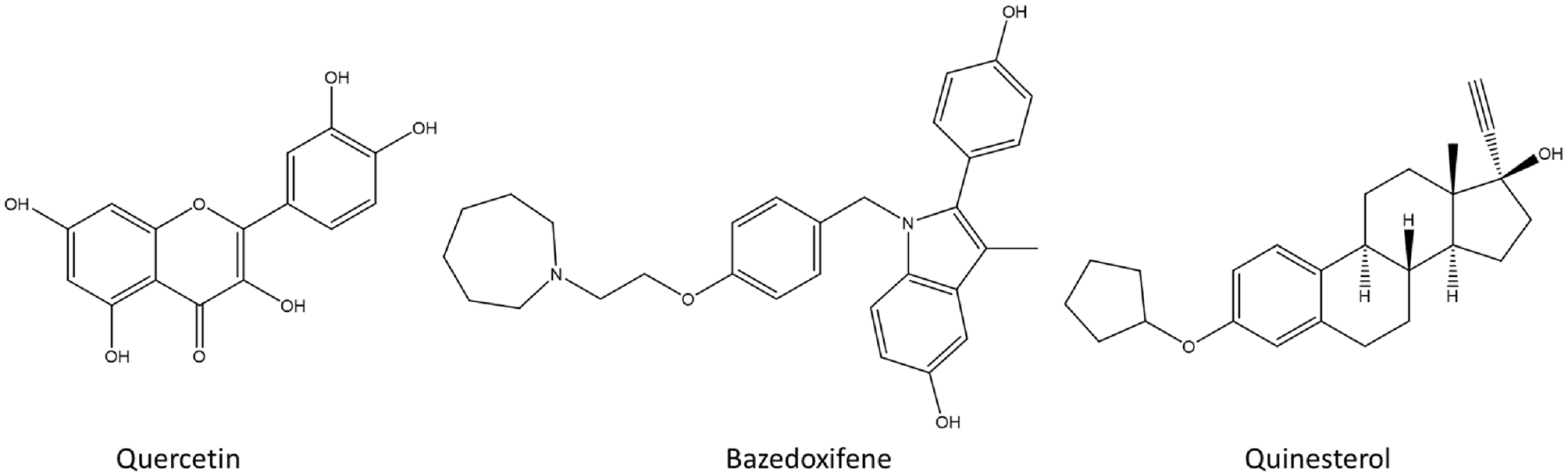
Chemical structures of quercetin, bazedoxifene, and quinestrol.

In Fig. 1, the chemical structures of the four estrogen molecules used in the docking study are displayed, depicting Tanimoto similarity scores ranging from 0.2782 to 0.6103. The docking results indicated that these molecules exhibited similar binding locations on the receptor surface and comparable binding energy values. Consequently, we focused on the complexes with estradiol as a representative estrogen and further examined them in detail. Among the docking results, poses with higher binding affinities than those with quercetin were selected, and bazedoxifene and quinestrol were chosen as representative prototypes (Fig. 2). Notably, estrogens demonstrated better docking scores compared to bazedoxifene and quinestrol in the case of the CD80 protein, and thus the results obtained with estrogens are also presented. Additional information on the binding modes of all other docking poses can be found in the supplementary information section.

### Docking studies of checkpoint proteins with estrogen receptor modulators (ERMs)

In this section, we present the results of docking ERMs to checkpoint proteins. Structurally, all these proteins are based on the immunoglobulin fold domain (IgV) architecture. The human checkpoint proteins CTLA-4, PD-1, and PD-L1, along with one of the ligands of CTLA-4, CD80, were used as docking targets for the calculations. Values of Tanimoto similarity between the studied estrogens and SERMs and known ligands of checkpoint proteins were used for assessing the relevance of the binding mode. Molecular docking calculations were performed using the AutoDock Vina software^25^ and 3-D crystallographic structures were obtained from the Protein Data Bank^26^. Only docking poses with a free energy of binding lower than -5.5 kcal/mol were considered, which corresponds to an approximate “interaction constant” value of 0.1 mM or lower. The figures of relevant of docking poses are showed in Fig. S1–Fig. S17.

#### Molecular docking of cytotoxic T-lymphocyte protein 4 (CTLA-4)

Considering the evidence of the effects of compounds from *Rhus verniciflua* Stokes on the CTLA-4/CD80 axis, we performed docking studies with both CTLA-4 and CD80^21^. The CTLA-4 receptor (Alpha Fold entry P164010-F1) shares a similar topological organization with the PD-1 protein. Its N-terminal side contains a single extra-cellular IgV fold domain, followed by a transmembrane helix connecting to the intracellular segment. The function of the protein is to bind its physiological ligands, CD80 and CD86. For structural analysis, we selected the 3D structures of CTLA-4 complexes with two proteins, PDB ID 1I8L and 1I85, respectively. The 3D structures of hCTLA-4 N-terminal domain complexes with monoclonal antibodies (mABs, PDB ID: 5TRU, 6RP8, 7SU0, 7SU1, 5XJ3, 5GGV, 6XY2, 6RQM, 7DV4) reveal that the blocking antibodies cover the CD80 and CD86 binding surface. The CTLA-4 residues directly involved in the interactions with CD80 and CD86 are listed in Table S1.

To generate search boxes, we utilized the structural model of the residues listed in the Supplementary Information (Tables S1 and S2). Docking was performed using the 17 SERMs listed in Table 1. The most significant docking scores are highlighted in red in the Table, which summarizes the docking scores and approximate Ki values for each SERM with CTLA-4 and hCD80.

**Table 1:** Results of the docking calculations for hCTLA-4 and hCD80.

SERM	hCTLA-4 domain (PDB ID 3OSK, chain B)		hCD80 (coordinates from PDB ID 1I8L, chain B)	
	Docking score (kcal/mol)	Approximate K_*i*_ (µM)	Docking score (kcal/mol)	Approximate K_*i*_ (µM)
Bazedoxifene	− 6.2	28.5	− 4.7	358
Clomifene	− 5.3		− 4.3	
Cyclophenyl	− 5.0		− 5.0	
Estradiol	− 6.2	28.5	− 6.7	12.25
Estrane	− 4.6		− 4.6	
Estriol	− 5.5	93	− 5.8	60
Estrone	− 5.9	47.3	− 7.1	6.24
Genistein	− 4.9		− 5.5	93
Genistin	− 5.3		− 5.6	78.46
Luteolin	− 5.3		− 5.9	47.29
Quercetin	− 5.4	110	− 5.4	110
Quinestrol	− 6.8	10.4	− 6.1	33.74
Raloxifene	− 5.8	56	− 5.4	
Ridaifen-b	− 5.2		− 5.0	
Tamoxifen	− 5.0		− 4.6	
Toremifene	− 4.8		− 4.5	
XL-147	− 6.1	33.8	− 5.5	93

**Figure 3:**
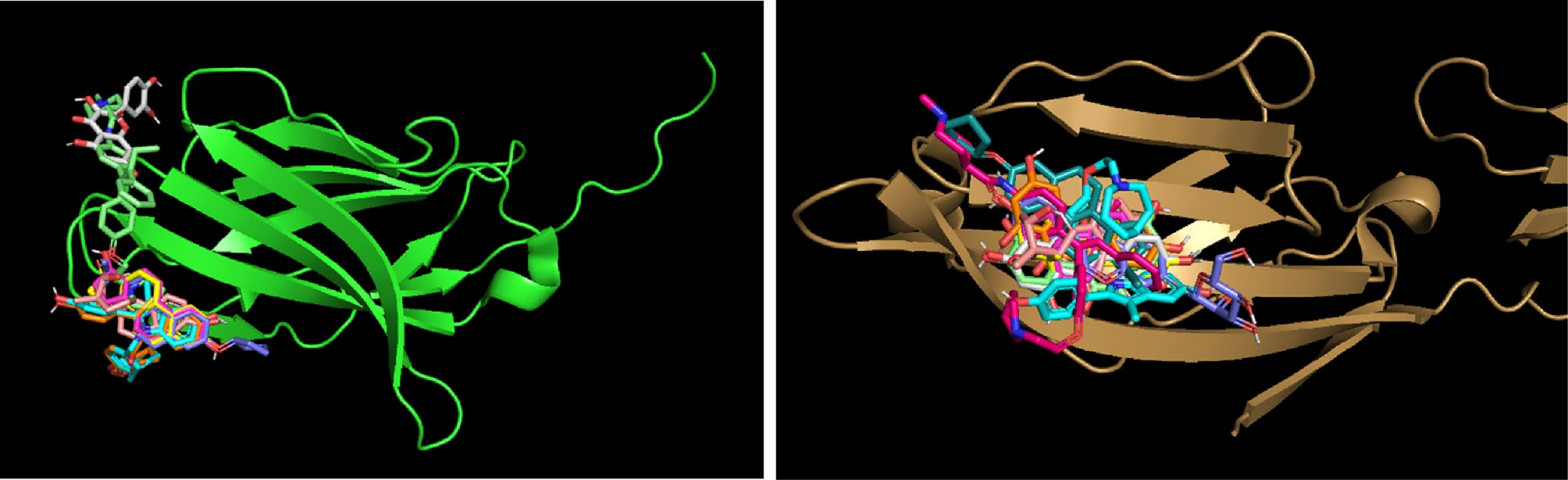
Docking locations of SERMs on the surface of CTLA-4 (green) and CD80 (bronze). The small molecules shown here are those with significant values of docking scores (Table 1).

For CTLA-4 (depicted in green), there is one docking location where most SERM molecules are predicted to bind. There is a second, less populated location where only two molecules are docked. In contrast, for CD80 (depicted in bronze), the docking calculations predict only one docking site (Fig. 3).

The molecular docking analyses of quinestrol, bazedoxifene, quercetin, estradiol, raloxifene, and XL-147 **(**Fig. S1–S5) with hCTLA-4 reveal their potential as inhibitors of CTLA-4 signaling through specific binding interactions to that protein. These findings provide insights into the binding modes and molecular recognition of these compounds with hCTLA-4. Overall, these docking studies shed light on the potential role of quinestrol, bazedoxifene, and quercetin as inhibitors of CTLA-4 signaling, opening avenues for further research in the development of novel therapeutics targeting immune checkpoint molecules.

**Figure 4:**
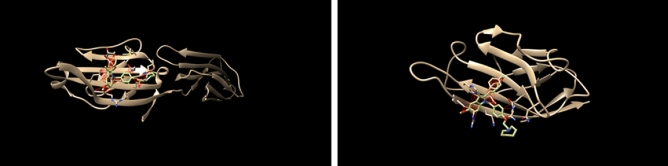
Steric clashes of CD80 and CD86 with bound bazedoxifene on CTLA-4.

In addition to the docking analysis, a steric clash analysis was conducted specifically for CTLA-4 to assess the potential interference of SERM binding with the physiological ligands CD80 and CD86. The analysis revealed extensive steric clashes between the docked SERMs and the binding sites of CD80 and CD86 on CTLA-4 (Fig. 4). Note that this steric clash analysis was specifically focused on CTLA-4 and was not performed for PD-1 or PD-L1. Given that the SERMs bind in the region of CTLA-4, PD-1, or PD-L1 that corresponds to the binding site of the physiological ligands, it is reasonable to expect similar steric clashes with the bound physiological ligands for PD-1 (such as PD-L1) as well.

Figure 4 illustrates the potential steric clashes between CD80 and CD86 ligands and bazedoxifene bound to CTLA-4. To generate the figures, a superposition operation was performed with CTLA-4, aligning the coordinates of CTLA-4 complexed with CD80 or CD86 to the location of CTLA-4 in the 3-D structure used for the docking (the B-subunit of PDB 3OSK). The atomic coordinates of CTLA-4 were then removed for clarity, and the steric contacts between the docked bazedoxifene and CD80 (left) or CD86 (right) are depicted as thin red lines. The extensive steric clashes observed between the docked bazedoxifene and CD80/CD86 suggest potential interference with the binding of these physiological ligands to CTLA-4. These findings highlight the potential inhibitory effects of bazedoxifene on the binding of CD80 and CD86, which are crucial for the immune response mediated by CTLA-4. Taken together, these findings highlight the potential interference of SERMs, such as bazedoxifene, with the binding of CD80 and CD86 to CTLA-4, which are crucial for the regulation of immune responses mediated by CTLA-4. The steric clashes observed emphasize the importance of further investigation to understand the implications of SERM binding for the functional interactions between immune checkpoint proteins and their physiological ligands.

#### Molecular docking of T-lymphocyte activation antigen CD80

CD80, one of the physiological ligands of CTLA-4, plays a crucial role in T-cell activation. It is a co-stimulatory molecule that delivers a second signal to T cells upon interaction with CD28^27^. Structurally, CD80 (AlphaFold entry P33681-F1) is a type 1 transmembrane protein expressed on the surface of antigen-presenting cells. Its extracellular N-terminal region consists of two sequential Ig fold domains, followed by a transmembrane helix and an intra-cytoplasmic carboxy-terminal segment. The primary function of CD80 is to enhance and sustain T-cell activation by binding to CD28. However, this activation process is inhibited when CD80 binds to CTLA-4, which outcompetes CD28. Consequently, the immune response is terminated. Given the inhibitory effect of compounds from *Rhus verniciflua* Stokes on the CTLA-4/CD80 axis^21^, conducting docking studies of ERMs with CD80 can provide valuable insights into the potential effects of these estrogens on the interaction between CTLA-4 and CD80.

By exploring the docking of ERMs to CD80, we aimed to further elucidate the impact of these estrogenic compounds on the CTLA-4/CD80 interaction and its downstream signaling. From the results presented in Table 2 and considering the chemical similarity between the four estrogens (with Tanimoto chemical similarity scores up to 0.61), it is highly likely that if these molecules possess the capability to bind to proteins within the CTLA-4/CD80 axis, their primary binding site would be CD80. The structural and functional characteristics of CD80 make it a potential target for these estrogens, as depicted in Figure S7. These findings support the notion that these four estrogens, due to their chemical similarity, would most likely interact with CD80, a key protein within the CTLA-4/CD80 axis. Moreover, the docking scores obtained for quercetin (Table 2, Figure S8) and quinestrol (Table 2, Figure S8) suggest their potential as inhibitors. The docking scores reflect the strength of the interaction between these compounds and the target protein, with lower scores indicating more favorable binding affinities. In the case of quercetin and quinestrol, the docking scores suggest a strong potential for inhibitory activity against the target protein associated with the CTLA-4/CD80 axis.

**Table 2:** Results of the docking calculations for the PD-L1 symmetric homodimer (N-terminal Ig fold domain).

E.R.M	Docking score (kcal/mol)	Approximate K_*i*_ (µM)
Bazedoxifene	− 10.7	14.3
Clomifene	− 7.9	
Cyclophenyl	− 8.6	
Estradiol	− 10.5	20.1
Estrane	− 6.5	
Estriol	− 7.5	
Estrone	− 10.2	33.4
Genistein	− 10.3	28.2
Genistin	− 10.4	23.8
Luteolin	− 10.0	46.8
Quercetin	− 9.5	108.7
Quinestrol	− 11.2	6.17
Raloxifene	− 10.5	20.1
Ridaifen-b	− 9.1	
Tamoxifen	− 7.8	
Toremifene	− 7.4	
XL-147	− 10.0	46.8

#### Molecular docking of programmed cell death 1 ligand 1 (PD-L1)

PD-L1 is a protein that is anchored in the plasma membrane through a single transmembrane helix (AlphaFold entry Q9NZQ7-F1). It serves as a ligand for the Programmed Cell Death Protein 1 (PD-1) receptor^28^.

##### Symmetric homo-dimer (N-terminal IgV fold domain)

Multiple 3D structures of the N-terminal domain of PD-L1 have been determined in a complex with non-peptide small molecules (PDB IDs 5J89, 5J8O, 5N2F, 5N2D, 5NIU, 6NM7, 6NOJ, 6NOS, 6R3K, 6RPG, 6NM8, 6VQN, 7DY7, 7BEA, 7NLD). These crystal structures reveal the PD-L1 domain as a symmetric homodimer, different from the skewed homodimer crystal structure of PD-L1 (a construct that contains the two N-terminal Ig fold domains in each monomer, PDB id 4Z18). The dimerization of PD-L1 is believed to be induced by the binding of small molecules^29^. Importantly, this homo-dimeric form of PD-L1, when bound to small molecules, undergoes internalization, resulting in its removal from the cell surface.

The symmetric homodimer configuration of PD-L1 exhibits a central channel between the two domains, which accommodates the binding of small molecules (Figure S18). The region encompassing this channel was utilized to define the search box for docking experiments. Prior to that, blind docking was also performed with the entire homo-dimer: all ERMs were docked either within the central channel or at the “base” of this channel, away from the N-terminus of the polypeptide chain (not shown).

Table 2 presents the results of the molecular docking calculations performed with the symmetric PD-L1 homodimer. The docking scores, representing the binding affinity, are provided in kcal/mol, while the approximate Ki values in µM give an indication of the “strength of binding.” These results reveal the interaction between each ERM and the homodimer. Notably, certain ligands such as bazedoxifene, estradiol, and quinestrol exhibited strong binding affinity with lower docking scores and Ki values, indicating a potential favorable interaction with this conformation of PD-L1. Detailed results concerning these three ligands are described in the following figures.

The Tanimoto similarity indices between the 17 SERMs investigated in this study and a representative ligand, derivative of Schiff base (called compound A, R81) observed bound to the homo-dimeric form of PD-L1^29^, indicate a relatively low chemical similarity, suggesting that these ERMs may not readily induce formation of a symmetric dimer. This finding raises caution regarding the potential restructuring of PD-L1 organization on the cell surface by the investigated SERMs. Previous studies have reported dissociation constants of 10.19 μM and 4.53 μM for PD-1 and PD-L1, respectively, in the presence of quercetin^22^. Similarly, kaempferol 7-O-rhamnoside showed dissociation constants of 31.1 μM and 19.7 μM. Although the 3D complex structures of these compounds with PD-1 and PD-L1 have not been determined, a reasonable hypothesis can be formulated that both quercetin and kaempferol bind to residues involved in the PD-1:PD-L1 interface, disrupting their interaction^23^. This suggests a potential for modulating the PD-1/PD-L1 axis by these compounds.

Based on the docking calculations for the symmetric homodimer PD-L1, the results suggest hypothetical binding of the ERMs, including quercetin, bazedoxifene, quinestrol, and others, to the central channel of the homodimer. However, it is important to note the low chemical similarity between the ERMs and the representative ligand together, with the absence of experimental complex structures, additional studies are needed to confirm the actual binding affinities and functional implications of these interactions. Overall, the docking results presented in Figures S10–S12 provide insights into the potential binding modes and interactions between the investigated ERMs and the hPD-L1 symmetric homodimer. However, caution should be taken when interpreting these findings, particularly in relation to the induction of the symmetric dimer formation by the ERMs. Future experimental investigations are warranted to establish the precise binding affinities, evaluate the impact on PD-L1 organization, and determine the functional consequences of these interactions.

##### PD-L1 N-terminal IgV fold domain

For the docking calculations, we utilized multiple 3-D structures of PD-L1 to gain a comprehensive understanding of the binding interactions. Firstly, we employed the structure of the PD-L1 domain present in the complex with PD-1 (PDB id 4ZQK, resolution of 2.5 Å) as a starting point. Additionally, high-resolution models of PD-L1 alone (PDB id 4Z18, resolution of 1.8 Å) and in complex with a macrocyclic inhibitor (PDB id 5O45, resolution of 0.99 Å) were also incorporated into the docking calculations. The utilization of higher resolution structures is expected to improve the accuracy of the docking calculations by providing more precise positional and geometric information, potentially revealing additional docking poses that may have been missed in calculations based on the lower resolution structures. The residues involved in the interaction with the human PD-1 IgV domain are listed in Table S3.

Table 3 presents the results of the docking calculations performed with the PD-L1 N-terminal domain and PD-1 N-terminal domain. These calculations provide insight into the potential binding locations of the ERMs on the surfaces of PD-L1 and PD-1.

**Table 3:** Results of the docking calculations for the PD-L1 N-terminal domain and PD-1 N-terminal domain.

E.R.M	PD-L1 N-terminal domain (PDB id 4ZQK, 4Z18, 5O45)		PDB ID	PD-1 N-terminal domain (PDB id 4ZQK or 6UMV)		PDB ID
	Docking score (kcal/mol)	Approximate K_*i*_ (µM)		Docking score (kcal/mol)	Approximate K_*i*_ (µM)	
Bazedoxifene	− 6.1	33.74	4ZQK	− 6.6	14.6	6UMV
Clomifene	− 5.5		5O45	− 5.2		6UMV
Cyclophenyl	− 6.3	24.07	5O45	− 6.0	40	6UMV
Estradiol	− 6.9	8.74	5O45	− 6.9	8.74	6UMV
Estrane	− 5.9		4Z18	None		
Estriol	− 6.5	17.17	5O45	− 6.9	8.74	6UMV
Estrone	− 6.5	17.17	5O45	− 6.8	10.35	6UMV
Genistein	− 6.5	17.17	5O45	− 6.2	28.5	6UMV
Genistin	− 6.9	8.74	5O45	None		
Luteolin	− 6.5	17.17	5O45	None		
Quercetin	− 6.3	24.07	4Z18	None		
Quinestrol	− 7.2	5.27	5O45	− 6.0	40	4ZQK
Raloxifene	− 6.8	10.35	5O45	− 6.4	20.33	6UMV
Ridaifen-b	− 6.0	40	5O45	None		
Tamoxifen	− 6.2	28.5	5O45	− 4.8		4ZQK
Toremifene	− 5.5		5O45	− 4.5		4ZQK
XL-147	− 7.0	7.38	5O45	− 6.0	40	6UMV

**Figure 5:**
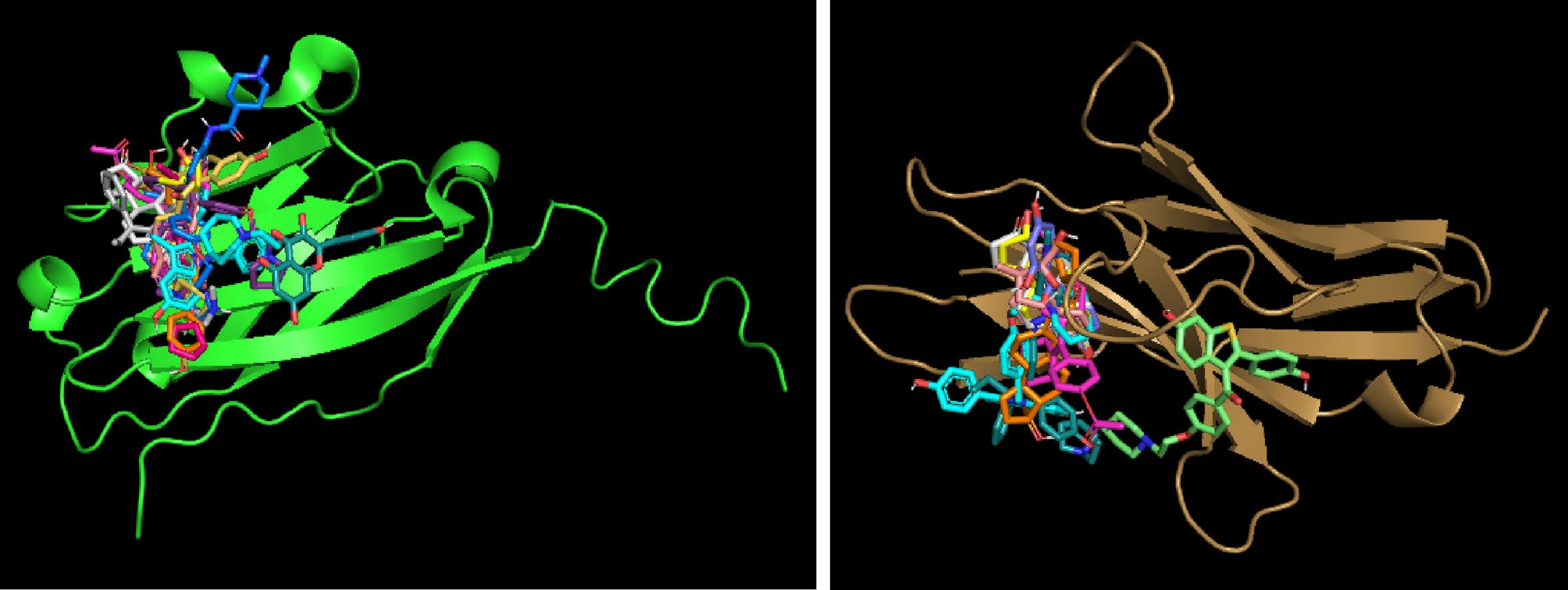
Docking locations of ERMs on the surface of PD-L1 (green) and PD-1 (bronze). The small molecules shown here are those with significant values of docking scores (Table 3).

Figure 5 illustrates the docking locations of the ERMs on the surfaces of PD-L1 (depicted in green) and PD-1 (depicted in bronze). The small molecules shown in the figure correspond to those with significant docking scores, as listed in Table 3.

In contrast to the binding predictions for CD80, the docking calculations for PD-L1 and PD-1 indicated potential binding sites on their surfaces where the majority of the ERMs are likely to bind (Fig. 5). Furthermore, for both proteins, the calculations suggest a second, less populated binding location where only one ERM is predicted to bind. The docking results presented in Figures S13, S14, and S15 provide insights into the potential binding modes and interactions between bazedoxifene, quercetin, and quinestrol, respectively, with the PD-L1 N-terminal IgV fold domain. These computational docking simulations reveal critical interactions between the ligands and the receptor, as depicted in the schematic and 2D diagrams.

The findings suggest potential binding modes and provide a visual representation of the interactions, highlighting the contributions of specific amino acid residues and their respective atoms or groups. Consolidating these results with the overall docking findings in this section, they collectively offer valuable insights into the binding preferences and interactions of the investigated ligands with the PD-L1 N-terminal IgV fold domain. As mentioned before, further experimental investigations are warranted to validate these findings and establish the precise binding affinities and functional consequences of these interactions. The docking results presented in this entire section provide valuable insights into the binding preferences and interactions of the investigated ligands with the PD-L1 N-terminal IgV fold domain. Bazedoxifene exhibited a docking score of − 6.1 kcal/mol, indicating a moderate binding affinity to PD-L1. The interactions involved key amino acid residues, including GLY 119, TYR 118, PHE 19, VAL 44, ALA 18, THR 20/22, GLU 45, LEU 94, and ASN 96. These findings suggest that bazedoxifene holds promise as a selective modulator of PD-1 activity. Similarly, quercetin demonstrated a docking score of − 6.3 kcal/mol, indicating a relatively strong binding affinity for PD-L1. Notable interactions involved VAL 42, LYS 46, ALA 52, GLY 119, PHE 42, PRO 43, GLU 45, ASP 49, and TYR 118. These findings suggest that quercetin may be capable of disrupting the interaction between PD-1 and PD-L1, potentially affecting the PD-1/PD-L1 axis. Remarkably, quinestrol exhibited potent binding affinities not only with PD-L1 but also with PD-1, CTLA-4, and CD80. The docking scores for quinestrol (− 7.2 kcal/mol for PD-L1, − 6.0 kcal/mol for PD-1, − 6.8 kcal/mol for CTLA-4, and − 6.1 kcal/mol for CD80) indicate strong and stable interactions with these proteins. This suggests that quinestrol has the potential to modulate the activity of PD-L1, PD-1, CTLA-4, and CD80 through its high binding affinity. Quinestrol may therefore represent a versatile therapeutic candidate or serve as a lead compound for further drug development targeting these proteins. For this reason, the case of quinestrol is discussed after the results of docking with the PD-1 N-terminal domain.

The findings presented in this section provide a foundation for future investigations into the role of the PD-L1 N-terminal IgV fold domain and its interaction with ligands, paving the way for potential therapeutic strategies targeting the PD-1/PD-L1 axis. In the following section, we will explore the binding characteristics and interactions of the investigated ligands with the PD-1 extracellular domain, shedding light on their potential as modulators of PD-L1 function.

#### Molecular docking of Programmed Cell Death protein 1 (PD-1)

The receptor protein PD-1 (AlphaFold entry Q15516-F1) is a key player in the PD-1/PD-L1 immune checkpoint pathway^30^. It shares a similar topological organization with its two ligands, PD-L1 and PD-L2. PD-1 consists of a single extracellular IgV fold domain located at the N-terminal side of its amino acid sequence, followed by a transmembrane helix that connects to the intracellular segment. Like PD-L1, PD-1 is anchored in the plasma membrane of T cells and pro-B cells. Through molecular docking simulations, we investigated the binding characteristics and interactions of the investigated ligands with the N-terminal IgV domain of hPD-1. The residues of the hPD-1 N-terminal IgV domain that form the interaction surface with the ligands hPD-L1 and hPD-L2 are summarized in Table S4. These findings provide insights into the potential modulation of PD-1 activity by the investigated ligands. In this section, we will present the docking results and discuss the binding preferences and interactions of the ligands with PD-1. These findings contribute to our understanding of the PD-1/PD-L1 axis. The docking simulation results revealed the binding interactions between PD-1 and bazedoxifene, as shown in Figure S16.

Bazedoxifene demonstrated a favorable docking score of − 6.6 kcal/mol, indicating its potential binding affinity for the PD-1 protein. The docking scores for quinestrol with the four proteins under investigation (− 7.2 kcal/mol for PD-L1, − 6.0 kcal/mol for PD-1, − 6.8 kcal/mol for CTLA-4, and − 6.1 kcal/mol for CD80) indicate strong and stable interactions with these proteins. This suggests that quinestrol has the potential to modulate the activity of PD-L1, PD-1, CTLA-4, and CD80 through its high binding affinity. Quinestrol may therefore represent a versatile therapeutic candidate or serve as a lead compound for further drug development targeting these proteins. The molecular docking analysis of Programmed Cell Death Protein 1 (PD-1) in the context of the PD-1/PD-L1 axis provides valuable insights into the binding preferences and interactions of potential modulators, namely bazedoxifene (Figure S16) and quinestrol (Figure S17). The results suggest that both bazedoxifene and quinestrol exhibit potential binding affinities for PD-1, indicating their potential as modulators of PD-1 activity.

### Molecular dynamics simulations of selected SERMs and estradiol against the PD-L1/PD-1 axis

The docking simulations provided valuable suggestions that the biological activity of estradiol and some SERMS (e.g., quercetin, bazedoxifene, and quinestrol) could be associated with inhibition of immune checkpoint inhibitors, especially for the PD-1/PD-L1 axis. Nevertheless, the accuracy of molecular docking may not always be sufficient. To obtain more accurate estimates of the free energies of binding, we used a more advanced method, molecular dynamics simulations. Using this method, we investigated the interactions of quercetin, bazedoxifene, estradiol, and quinestrol with both PD-L1 and PD-1. The molecular dynamics simulations were performed using CHARMM, together with the CHARMM-GUI web server for input generation^31,32^. The results obtained using this procedure are shown in Tables 4 and 5**.**

**Table 4:** Free energy of binding (Δ-G, kcal/mol) to PD-L1 (5O45).

Ligand	Vina	Equilibration	Molecular dynamics
Quercetin	− 6.3	− 7.01	− 6.53
Bazedoxifene	− 6.1	− 7.64	− 7.28
Estradiol	− 6.9	− 7.49	− 7.45
Quinestrol	− 7.2	− 7.91	− 7.86

**Table 5:** Free energy of binding (Δ-G, kcal/mol) to PD-1 (6UMV).

Ligand	Vina	Equilibration	Molecular dynamics
Bazedoxifene	− 6.6	− 8.36	− 6.63
Estradiol	− 6.9	− 7.61	− 6.69
Quinestrol	− 6.0	− 8.06	− 7.55

The values of the free energies of binding obtained after both the initial equilibration step and the molecular dynamics simulations are significantly lower than those obtained from Vina docking, except for the binding of estradiol to PD-1. It should be noted, however, that molecular dynamics simulations address the dynamics of the ensembles, and the coordinates after MD show ligands slightly displaced from the lowest energy state. Even though the value of the binding energy calculated for the estradiol interaction with PD-1 was lower than in the case of molecular docking, its value was still significant. While the docking calculations provide valuable predictions, they are based on computational models with inherent limitations. Validation through additional experimental studies is crucial to confirm the binding affinities and functional implications. The integration of 3-D crystallographic structures, manual preparation using UCSF Chimera, docking with AutoDock Vina, and visualization with UCSF Chimera and BIOVIA Discovery Studio Visualizer enhances the reliability and comprehensibility of the results. These techniques contribute to a more comprehensive understanding of the molecular interactions, driving further research in the field. These findings suggest potential modulatory effects of quercetin, bazedoxifene, estradiol, and quinestrol on the activities of immune checkpoint inhibitors, at least on the PD-1/PD-L1 axis.

## Discussion

The above findings suggest that quercetin, bazedoxifene, estradiol and quinestrol could potentially be used as immune checkpoint inhibitors (ICIs), or at least that docking studies could contribute to better understanding of their biological/therapeutic effects. ICIs (e.g., α-PD-1, α-PD-L1, and α-CTLA-4) stimulate expansion of active immune cell subsets, e.g. those involved in T cell-mediated immune response. Quercetin and bazedoxifene (potential inhibitors of PD-L1 and PD-1, respectively) could block the interaction between TREGs, or tumor cells and T cells, leading to their inactivation and to apoptosis^10^ The interaction of CTLA-4 with CD80 represses the activity of macrophages and dendritic cells. Besides, quinestrol exhibits significant docking scores against PD-1, PD-L1, CTLA-4, and CD80. Hence, its application may result in strong activation of the anticancer immune system. Along these lines, dual immunotherapies are emerging as promising therapeutic approaches^33,34^. For example, the combination of α-PD-1 and α-CTLA-4 therapies can enhance the treatment efficacy with acceptable levels of adverse effects. In addition, in the case of patients with low expression of PD-L1, α-CTLA-4 might also decrease the risk of resistance against α-PD-1^34^. However, as discussed below, it is important to consider additional factors when assessing the potential of selected inhibitors (quinestrol, quercetin, and bazedoxifene) for therapeutic use.

Quercetin, for instance, is a commonly used dietary supplement known for its safety profile^35^, suggesting its potential for long-term use as an immune checkpoint inhibitor. Similarly, bazedoxifene has been reported to have low toxicity^36^. Nevertheless, it is worth noting that the application of quinestrol has been associated with potential risks, such as oxidative stress^37^ or allergic reactions^38^. Therefore, careful evaluation of the benefits and risks is necessary before considering quinestrol as a therapeutic agent. Furthermore, it is important to recognize the complexity of the modulation of PD-L1/PD-1 and CTLA-4/CD80 activities, as these processes can be influenced by various factors and signaling pathways. For example, the interactions between immune checkpoints and signaling pathways involving estrogen receptors or epidermal growth factor receptor (EGFR)^39,40^, as well as cytokines such as interleukin 6 (IL-6)^41^, play significant roles. It is important to note that some of the compounds used in the docking studies, such as quercetin and bazedoxifene, are direct inhibitors of EGFR^42^ and IL-6R^43^, respectively. Therefore, the potential interplay between immune checkpoints, EGFR, ER signaling, and the broader biological activities of the docking compounds should be carefully considered.

The EGFR family comprises transmembrane tyrosine kinases, including EGFR1/ErbB1 (also known as Her1), Her2/ErbB2, Her3/ErbB3, and Her4/ErbB4^44^. These receptors can be activated by various ligands such as EGF, transforming growth factor alpha (TGF-α), amphiregulin, betacellulin, heparin-binding EGF-like growth factor, epiregulin, epigen, and neuregulins 1–6^45^. EGFRs can also form active heterodimers with other ErbBs. Among them, HER2 is a preferred dimerization partner for the family members, although it does not have any known ligand^46,47^.

On the one hand, EGFR signaling induces glycosylation of PD-L1, which prevents its proteasome degradation. Non-glycosylated forms of PD-L1 are susceptible to degradation by GSK3β, which induces PD-L1 degradation^48^. Moreover, EGFR can stimulate the expression of PD-L1 through multiple pathways^49^, including as PI3K/AKT/mTOR^50^, IL-6/JAK/STAT3^41^ and NF-κB pathway^51^.

On the other hand, PD-L1 can strongly affect EGFR signaling^52^. Patients with pulmonary adenocarcinoma carrying EGFR mutations exhibit lower objective response rates and progression-free survival^16^. EGFR-TKI-resistant PC9 cells show elevated expression of YAP (a positive regulator of PD-L1 expression in the Hippo pathway) and PD-L1 compared to parental PC9 adenocarcinoma cells^53^. Knockdown of PD-L1 reduces cell proliferation and migration in EGFR-TKI-resistant PC9 cells^54^. In addition, EGFR-mutant NSCLC (Asian most prevalent molecular subtype) displays poor response to anti PD-1/PD-L1 treatment. However, gefitinib (tyrosine kinase inhibitor) reduces PD-L1 expression and IL-6 production in EGFR mutant cells^41^, which correlates with the dephosphorylation of signal transducer and activator of transcription 3 (STAT3; another direct activator of PD-L1 expression)^55^. Notably, several high-impact publications have suggested that combination therapy targeting both EGFR and PD-L1 exhibits potent synergic effects. In a competitive binding assay, a bispecific EGFR and PD-L1 antibody demonstrated an IC_50_ value approximately 140 lower than that of MockxPD-L1^56^. Preincubation of A431 cells with mAb 425 increased the IC_50_ value from 0.013 to 0.549 mg/ml. In EGF-treated MDA-MB-231 cells, the bispecific antibody strongly suppressed the EGFR signaling pathway^57^. In a mouse model, the tumor volume was significantly reduced. However, Chen et al. reported that in colon cancer cells, unlike insulin, EGF did not increase PD-L1 expression but supported its membrane localization^58^. In another mouse model, EGFR-driven lung tumors exhibited elevated levels of transforming growth factor beta-1 (TGF-β1), PD-1, and FOxp3 +^59^.

Estrogen receptors (ERαs and ERβs) are encoded by the estrogen receptor 1 (ESR1) and estrogen receptor 2 (ESR2) genes. They are expressed in normal mammary glands as well as in breast tumors^60,61^. Upon interaction with ligands such as estrogens and SERMs, ERs undergo conformational changes, leading to receptor dimerization and binding to specific DNA sequences called “Estrogen Response Elements” (EREs). The DNA binding domains of ESR1 and ESR2 share high similarity (96% homology), and both ERα and ERβ can interact with the majority of EREs. Additionally, ERs can regulate cellular signaling through nongenomic mechanisms. For example, ERα can activate the PI3K/Akt pathways^62^ and histone deacetylase 6, resulting in tubulin deacetylation^63^. Antiestrogens and SERMs have been shown to decrease the risk of developing prostate cancer or suppress metastasis in prostate cancer patients with high levels of ERβ expression^64,65^. However, their application can also have problematic effects. In ERα-positive breast cancer, prolonged estrogen deprivation can stimulate c-MYC, nuclear factor-κB (NF-κB), and hypoxia-inducible factor 1α (HIF-1α), leading to antiestrogen resistance^66^. In Hela cells, estrone (but not estradiol) support TNF-α-induced NF-KB signaling and epithelial-mesenchymal transition (EMT)^67^. Conversely, in ERα-positive MCF-7 breast cancer cells, estradiol stabilizes PD-L1 via the PI3K/Akt pathway, thereby increasing PD-L1 expression^68^. Treatment with estradiol in cocultured T cells decreases the expression of IFN-γ and IL-2 in Jurkat or primary T cells. Estradiol can induce a suppressive M2 macrophage phenotype and reduce the cytotoxic activity of NK cells^69,70^. In TREGs, estradiol stimulation leads to increased PD-1 levels and promotes their differentiation^71^.

The above suggests that modulation of the PD-1/PD-1L axis may be related to the effect of estrogens or SERMs on ER signaling. Nevertheless, the regulation of the PD-1/PD- 1L axis is a complex process, and the role of individual receptors and signaling factors is not easy to quantify. Overstimulation of the PI3K/Akt or JAK/STAT pathway plays an important role in this mechanism^72–76^. Depending on the conditions, used animal models and cells or studied patients, the high PI3K/Akt or JAK/STAT activity can be associated with various signaling molecules such as estrogens, IL-6, or EGF^16,71,77^. On the other hand, in human leukemic cells, the PD-1/PD-L1 blockade may activate PI3K/Akt/mTOR signaling, resulting in the loss of treatment efficacy^78^. In mast cells, PD-1 antibody can release histamine and cytokines (CCL2, TGF-β, TNF-α and VEGF) via the PI3K/AKT pathway^79^.

Estrogen signaling could play a significant role in the dysregulation of CTLA-4. TGF-β1, produced by TREG cells, can upregulate the expression of PD-1 and CTLA-4 on T cells^80^. Several studies strongly suggest a crosstalk between estrogen and TGF-β signaling^81^. For instance, estrogen-activated ERα can form a complex with Smurf (ubiquitin ligase) and Smad, which is subsequently ubiquitinated and degraded^82^. However, estradiol has been shown to increase TGFβ1 secretion and promote neutrophil infiltration into MCF-7 cell mammospheres^83^, with similar results observed in mouse models. In this context, it should be mentioned that ICIs can display sex-dependent effects^84^. In the case of NSCLC patients, a higher number of CD4 + T cell counts, higher CD4/CD8 ratios, and their cytotoxic activity were observed in females than in age-matched males. However, the higher efficiency of the female immune system leads to development of more complex and redundant mechanisms of resistance such as higher expression of immune checkpoint molecules with inhibitory functions. Assuming that estrogens are inhibitors of both the PD-1/PD-1L and CTLA-4/CD80 axes and are inductors of their expression, this dual nature could at least partially help to clarify the sex-dependent difference in the effectivity of the immune system.

The aforementioned findings indicate that SERMs can regulate the activity of immune checkpoints via ERs. Moreover, high-impact studies have reported that certain SERMs can target other oncogenic signaling pathways and factors associated with the activity and expression of immune checkpoints. For example, quercetin is a potent inhibitor of various oncogenic pathways^84^, as shown by the calculated free energy of binding with potential partners (Table S5)^42,85–89^. In prostate cancer cells, quercetin has been found to reverse EGF-induced EMT and invasiveness through inhibition of the EGFR/PI3K/Akt pathway^90^. Quercetin also significantly decreases the levels of HSP27 mRNA^91^; Hsp27 supports IkBα degradation, a repressive factor in NF-kB signaling^92^. QFJDD (a natural agent containing quercetin, luteolin, kaempferol, wogonin, baicalein, and acacetin) downregulates PD-L1 expression in the mouse model of Lewis lung carcinoma by regulating HIF-1α, EGFR, JUN, and NF-κB signaling pathways ^89^. Bazedoxifene and raloxifene are known direct inhibitors of gp130 (also called IL-6Rβ) and have been shown to repress IL-6 signaling in various models^43,93–96^. Additionally, Song et al.^97^ reported that bazedoxifene could act as an inhibitor of TNF-α signaling. In PANC1 pancreatic cancers cells, raloxifene nanoformulation leads to the downregulation of NF-kB and Bcl-2^98^. Raloxifene also exhibits strong inhibitory activity against histone lysine-specific demethylase 1, with an IC_50_ value of 2.08 μM^99^. Estrone and estrone sulfate have been identified as inhibitors of aldehyde oxidase, with IC_50_ values of 0.18 μM and 258 μM, respectively^100^. Similarly, raloxifene and bazedoxifene have been shown to have IC_50_ values of 0.028 μM and 0.19 μM, respectively. Aldehyde oxidase, a phase I drug-metabolizing enzyme, plays a significant role in the biotransformation of numerous drugs and xenobiotics, including oxidations of azaheterocycles and aldehydes, amide hydrolysis, and various reductions^101,102^.

## Conclusions

In conclusion, this report provides valuable insights into the possible role of SERMs and estrogens in modulating immune checkpoints, specifically CTLA-4, PD-L1, and PD-1. The results obtained from molecular docking and especially molecular dynamics simulations strongly suggest that SERMs (e.g., bazedoxifene, an approved drug supplement and quercetin, an approved food supplement) may function as inhibitors of immune checkpoint signaling, at least for the PD-L1/PD-1 axis. Revealing the role of SERMs and estrogens in the control of immune checkpoints is an interesting and challenging area of research. Understanding the regulatory mechanisms of immune checkpoint proteins, which critically influence immune responses, is of high importance for the development of future immunotherapy approaches.

## Materials and methods

3-D crystallographic structures were obtained from the Protein Data Bank^26^ to initiate the docking calculations. Prior to docking, the models were manually prepared to ensure accuracy by removing redundant conformations, crystallographic waters, ligands, and other irrelevant components. UCSF Chimera, a software known for its capabilities in handling molecular structures, was utilized for this purpose. Molecular docking calculations were performed using the AutoDock Vina software^25^. The calculations were conducted using the recommended parameters provided by the software authors to ensure consistency and accuracy. To visualize and analyze the docking results, three software tools were employed. UCSF Chimera was used to generate overall views of the docking outcomes^103^, while BIOVIA Discovery Studio Visualizer was utilized to create 2D diagrams and illustrate the interactions with amino acids^104^. PyMOL was employed to verify the positioning of the ligand on the receptor surface. In addition, Tanimoto chemical similarity scores were computed using the ChemMine tools web server^105^. These scores serve as a measure of the chemical similarity between different molecules, providing insights into their structural relationships.

All molecular dynamics simulations were performed using CHARMM^31^, together with the CHARMM-GUI web server for input generation^32^. Prior to performing the simulations, energy minimization of the coordinates of the docked ERM was carried out using the Yasara energy minimization server^106^. The coordinates of the PD-L1 and PD-1 complexes with the ERMs (quercetin, bazedoxifene, estradiol, and quinestrol), enclosed in an orthorhombic box containing water molecules plus K + and Cl- counterions, were first subjected to the standard equilibration protocol, followed by a 1 ns molecular dynamics run. The free energies of binding of the ligand were calculated after each step of the CHARMM procedure using the Prodigy web server^107^. The coordinate files used for the MD simulations are suitably modified PDB 5O45 and 6UMV entries for PD-L1 and PD-1, respectively^34,84^.

### Supplementary Information

Supplementary Information.

## Data Availability

The datasets used and/or analyzed during the current study are available from the corresponding author on reasonable request.
