# Supplementary material for: Investigation of the potential effects of estrogen receptor modulators on immune checkpoint molecules
Source: Sci Rep. 2024 Feb 6;14:3043. doi: 10.1038/s41598-024-51804-2 (PMC10847107; doi:10.1038/s41598-024-51804-2)
Supplement: Supplementary file 1 — Supplementary Information. [file 41598_2024_51804_MOESM1_ESM.docx]

**Investigation of the Potential Effects of Estrogen Receptor Modulators on Immune Checkpoint Molecules – Supplementary Information**

**Context>**

Table S1: List of residues forming the interaction surface with **hCD80** and **hCD86**.

Table S2: List of residues forming the interaction surface between **hCD80** and **hCTLA-4.**

Table S3: List of residues involved in the interaction with the **hPD-1** IgV domain.

Table S4: List of residues of the N-terminal **IgV** domain of **hPD-1** forming the interaction surface with ligands **hPD-L1** and **hPD-L2**.

**Figure S1**. Docking pose and interactions of quinestrol with hCTLA-4.

**Figure S2**. Docking pose and interactions of bazedoxifene with hCTLA-4

**Figure S3**. Docking pose and interactions of quercetin with hCTLA-4.

**Figure S4.** View of the docking pose of estradiol on the surface of hCTLA-4

**Figure S5.** View of the docking pose of raloxifene on the surface of hCTLA-4

**Figure S6.** View of the docking pose of XL-147 on the surface of hCTLA-4

**Figure S7.** View of the docking pose of estradiol on the surface of CD80

**Figure S8.** View of the docking pose of quercetin on the surface of CD80

**Figure S9.** View of the docking pose of quinestrol on the surface of CD80

**Figure S10.** View of the docking pose of bazedoxifene on the surface of the hPD-L1 symmetric homodimer

**Figure S11.** View of the docking pose of quercetin on the surface of the hPD-L1 symmetric homodimer

**Figure** **S12.** View of the docking pose of quinestrol on the surface of the hPD-L1 symmetric homodimer

**Figure S13.** Docking pose of bazedoxifene on the surface of hPD-L1

**Figure S14.** Scheme depicting the location of quercetin on the surface of the PD-L1 N-terminal IgV fold

**Figure S15.** Scheme of the location of quinestrol on the surface of the PD-L1 N-terminal IgV fold domain

**Figure S16.** Scheme of the location of bazedoxifene on the surface of PD-1

**Figure S17.** Scheme of the location of quinestrol on the surface of PD-1

**Figure S18.** Representation of the PD-L1 symmetric homodimer with the central channel

**Table S1: List of residues forming the interaction surface with hCD80 and hCD86.**

| hCD80 (1I8L) | GLU 33 – ARG 35 |  | GLU 97 – LEU 106 |
| --- | --- | --- | --- |
| hCD86 (1I85) | GLU 33 – ARG 35 | THR 53 | MET 99 – TYR 105 |
|  | GLU 33 – ARG 35 | THR 53 | GLU 97 – LEU 106 |

**Table S2:** **List of residues forming the interaction surface between hCD80 and hCTLA-4.**

| ARG 29 – MET 38 | MET 43 | VAL 83 – LEU 85 | PHE 92 – ARG 94 | LEU 97 |
| --- | --- | --- | --- | --- |

**Table S3: List of residues involved in the interaction with the hPD-1 IgV domain.**

| ALA 18 – ASP 26 | ILE 54 – GLU 58 | GLN 66 | ARG 113 – ARG 125 |
| --- | --- | --- | --- |

**Table S4: List of** **residues of the N-terminal IgV domain of hPD-1 forming the interaction surface with the ligands hPD-L1 and hPD-L2.**

| hPD-L1 (4ZQK) | VAL64-TYR68 | SER73-LYS78 | GLU84 | GLY124-GLU136 |
| --- | --- | --- | --- | --- |
| hPD-L2 (6UMT) | SER62-TYR68 | SER73-ALA80 | GLU84 | GLY124-GLU136 |
| composite | SER62-TYR68 | SER73-ALA80 | GLU84 | GLY124-GLU136 |

**Table S5: Values of calculated binding energy for quercetin binding to oncogenic proteins**

| **Target** | **Binding energy** | **Ref.** |
| --- | --- | --- |
| **EGFR** | −8.9 kcal/mol | [1] |
|  | −6.8 kcal/mol | [2] |
|  | −14.6 kj/mol | [3] |
|  | −15.1 kcal/mol | [4] |
|  | − 5.25 kcal/mol | [5] |
|  | -8.8 kcal/mol | [6] |
| **AKT** | −7.2 kcal/mol | [1] |
|  | -13.6kj/mol | [3] |
|  | −15.8 kcal/mol | [4] |
|  | -10.4 kcal/mol | [6] |
| **IL-6** | -4.7 kcal/mol | [5] |
| **mTOR** | −22.9 kcal/mol | [4] |
| **VEGFA** | -15.2 kj/mol | [3] |
|  | − 6.47 kj/mol | [5] |
| **Hsp90AB1** | −15.4 kj/mol | [3] |
| **H1F1A** | −22.9 kj/mol | [4] |
| **NFKBIA** | -7.0 kcal/mol | [6] |

**Molecular docking**

The figures in this section are presented in tabular form, displaying the top docking pose of ERMs on the surfaces of the outermost extra-cellular domains of the CTLA-4, PD-L1 and PD-1 receptors, and of CD80. The right side of the tables provides information on the amino acids involved, the type of interactions, and the relevant atoms/functional groups engaged in the interaction.

**hCTLA-4**

**
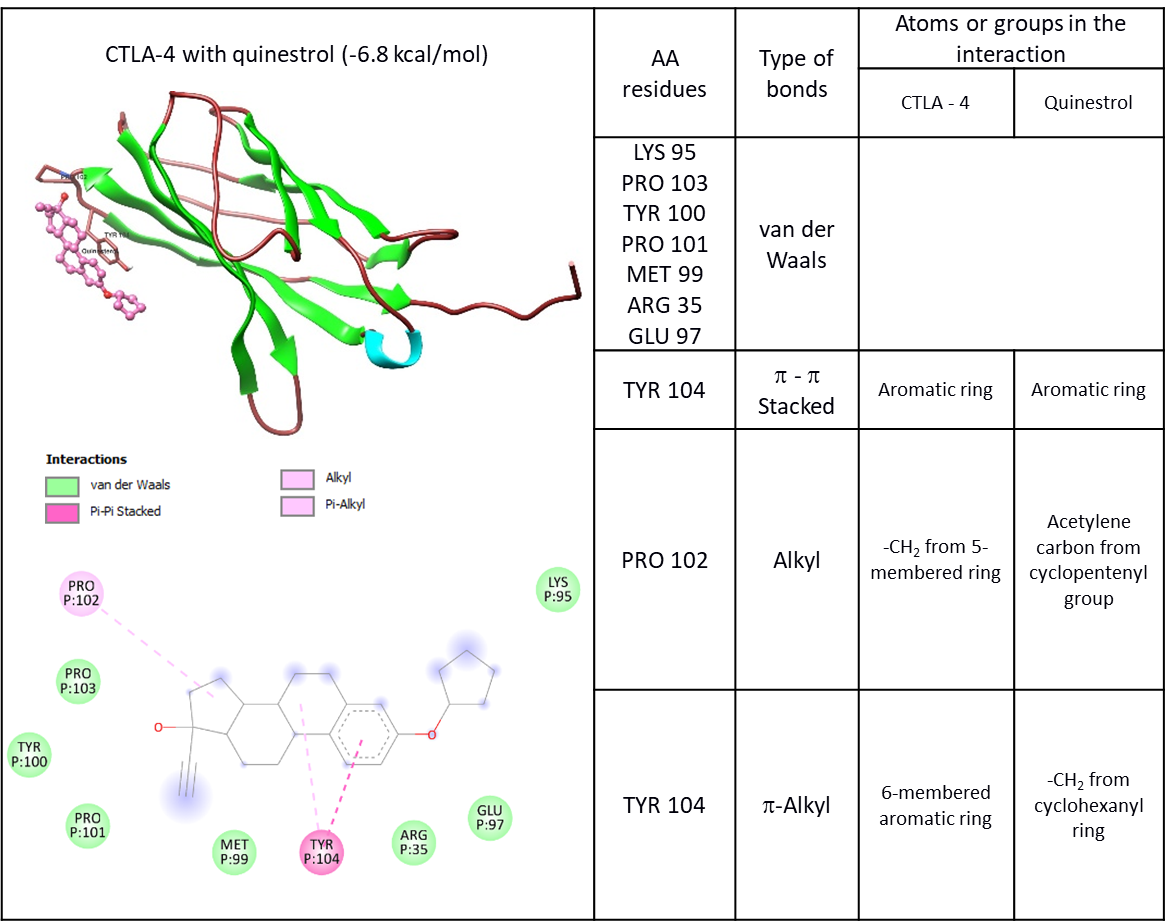
**

**Figure S1.** Docking pose and interactions of quinestrol with hCTLA-4. (Top left) Three-dimensional view of the docking pose of quinestrol on the surface of hCTLA-4, highlighting the ligand-receptor binding. (Bottom left) Schematic 2D diagram illustrating the specific interactions between quinestrol and the receptor. (Right panel) List of interactions between quinestrol and key amino acid residues in hCTLA-4.

**
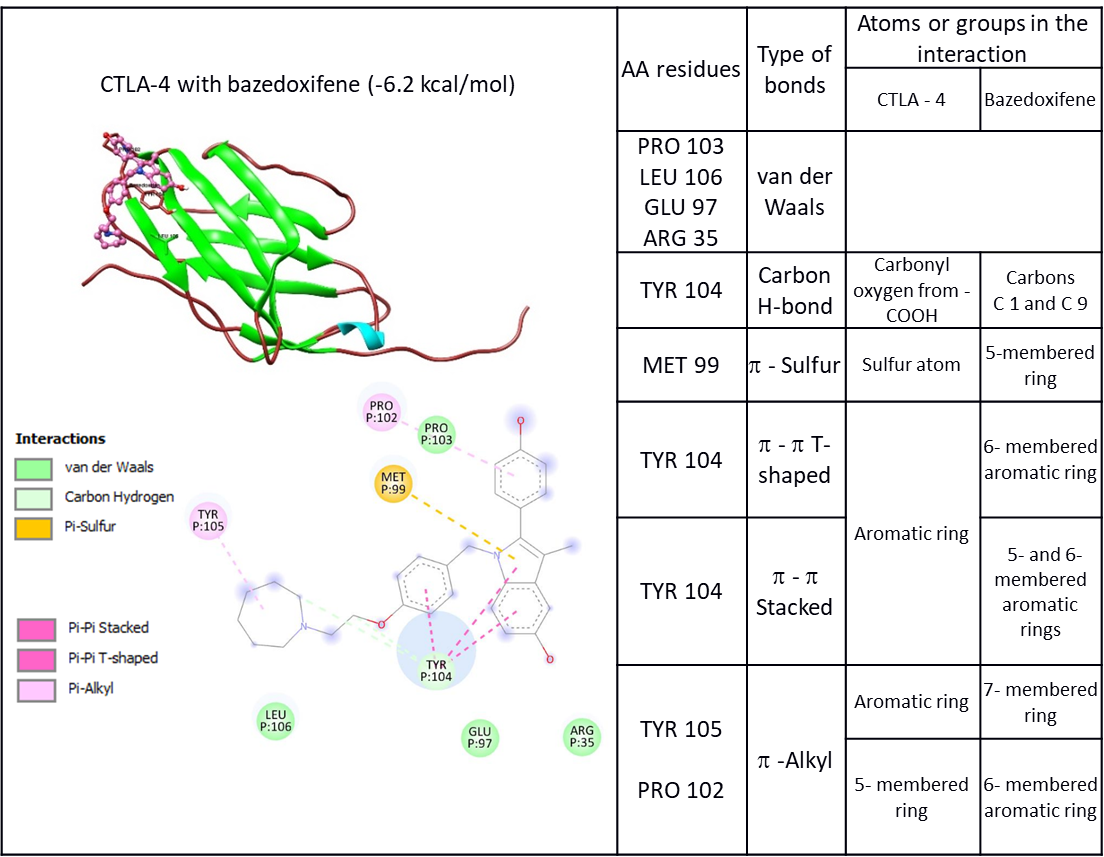
**

**Figure S2**. Docking pose and interactions of bazedoxifene with hCTLA-4. (Top left) Three-dimensional view of the docking pose of bazedoxifene on the surface of hCTLA-4, highlighting the ligand-receptor binding. (Bottom left) Schematic 2D diagram illustrating the specific interactions between bazedoxifene and the receptor. (Right panel) List of interactions between bazedoxifene and key amino acid residues in hCTLA-4.

**
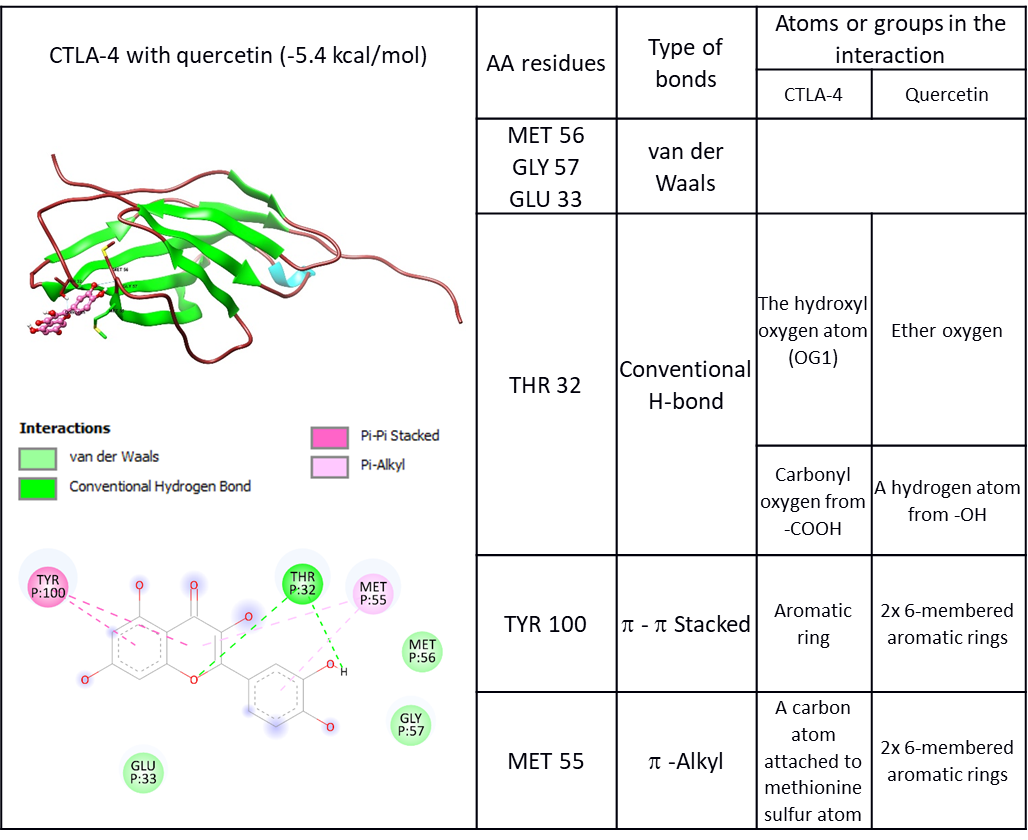
****Figure S3.** Docking pose and interactions of quercetin with hCTLA-4. (Top left) Three-dimensional view of the docking pose of quercetin on the surface of hCTLA-4, highlighting the ligand-receptor binding. (Bottom left) Schematic 2D diagram illustrating the specific interactions between quercetin and the receptor. (Right panel) List of interactions between quercetin and key amino acid residues in hCTLA-4.

**Figure S4.** View of the docking pose of estradiol on the surface of the hCTLA-4 (top left), schematic 2D diagram of the interactions with the receptor (bottom left). The right panel lists the interactions.

**Figure S5.** View of the docking pose of raloxifene on the surface of the hCTLA-4 (top left), schematic 2D diagram of the interactions with the receptor (bottom left). The right panel lists the interactions.

**Figure S6.** View of the docking pose of XL-147 on the surface of the hCTLA-4 (top left), schematic 2D diagram of the interactions with the receptor (bottom left). The right panel lists the interactions.

CD-80

**
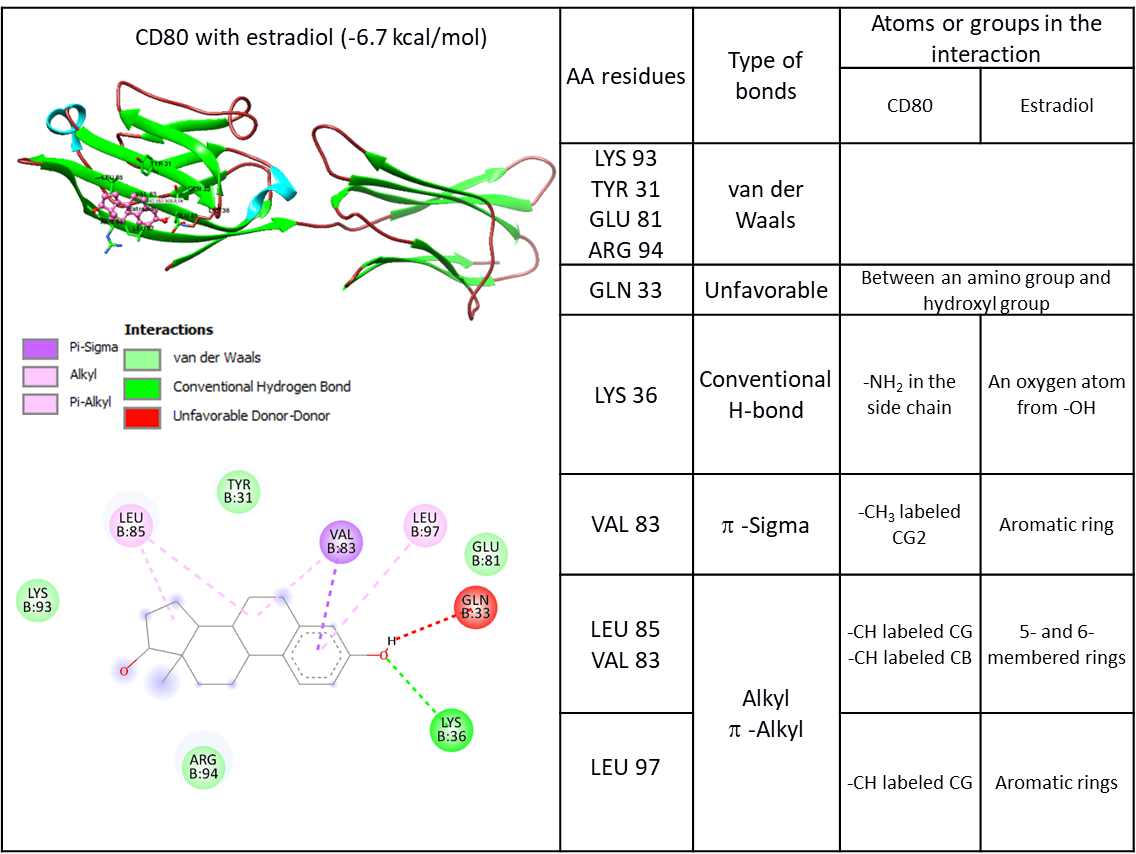
****Figure S7.** View of the docking pose of estradiol on the surface of CD80 (top left), schematic 2D diagram of the interactions with the receptor (bottom left). The right panel lists the interactions.

**
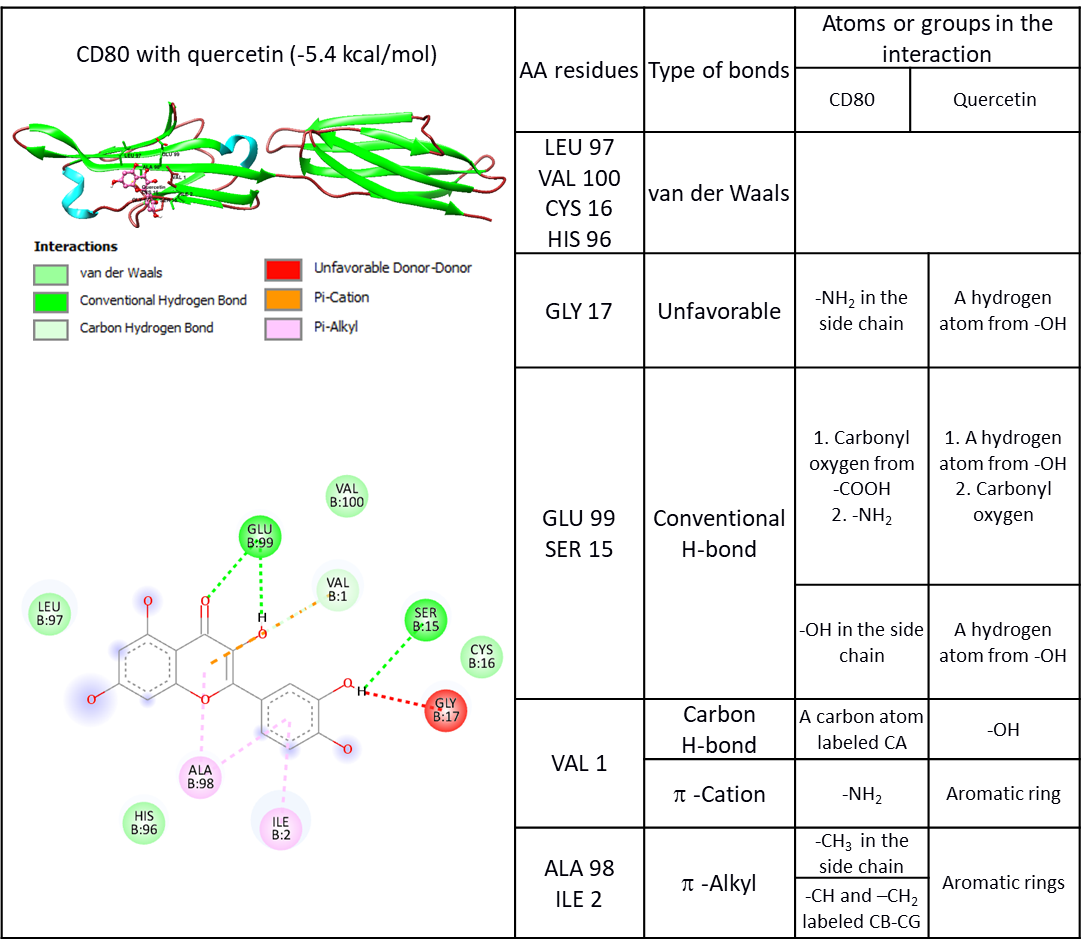
**

**Figure S8.** View of the docking pose of quercetin on the surface of CD80 (top left), schematic 2D diagram of the interactions with the receptor (bottom left). The right panel lists the interactions.

**
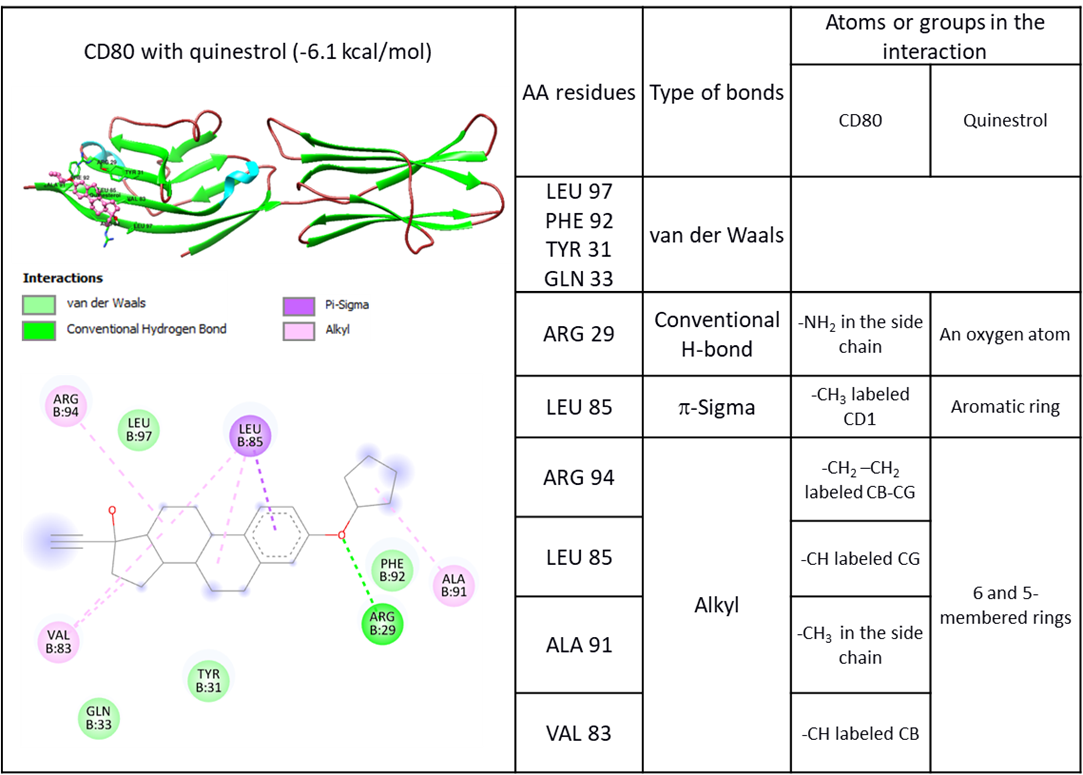
**

**Figure S9.** View of the docking pose of quinestrol on the surface of CD80 (top left), schematic 2D diagram of the interactions with the receptor (bottom left). The right panel lists the interactions.

**hPD-L1 symmetric homodimer**

**
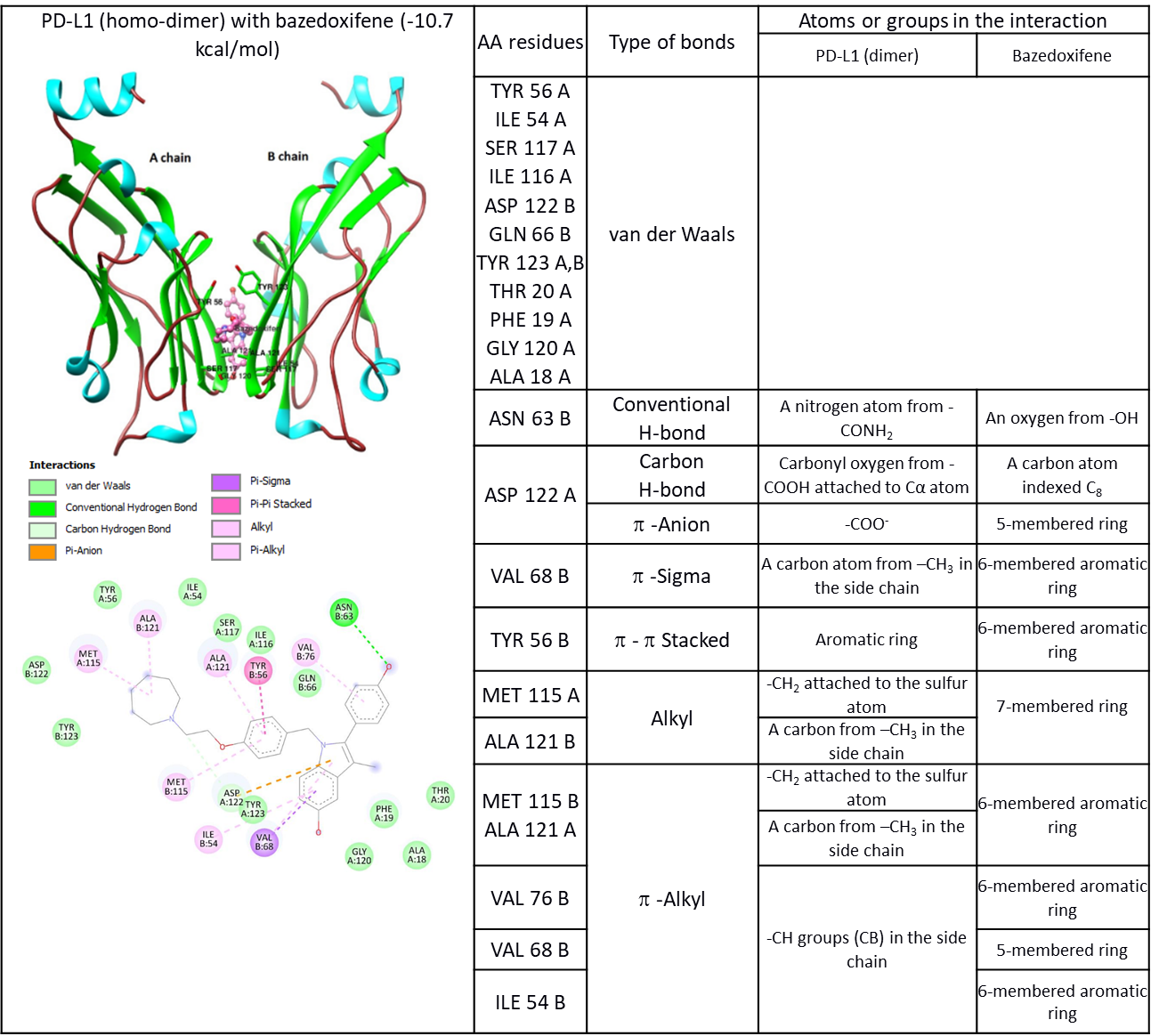
**

**Figure S10.** View of the docking pose of bazedoxifene on the surface of the hPD-L1 symmetric homodimer (top left), schematic 2D diagram of the interactions with the receptor (bottom left). The right panel lists the interactions.

**
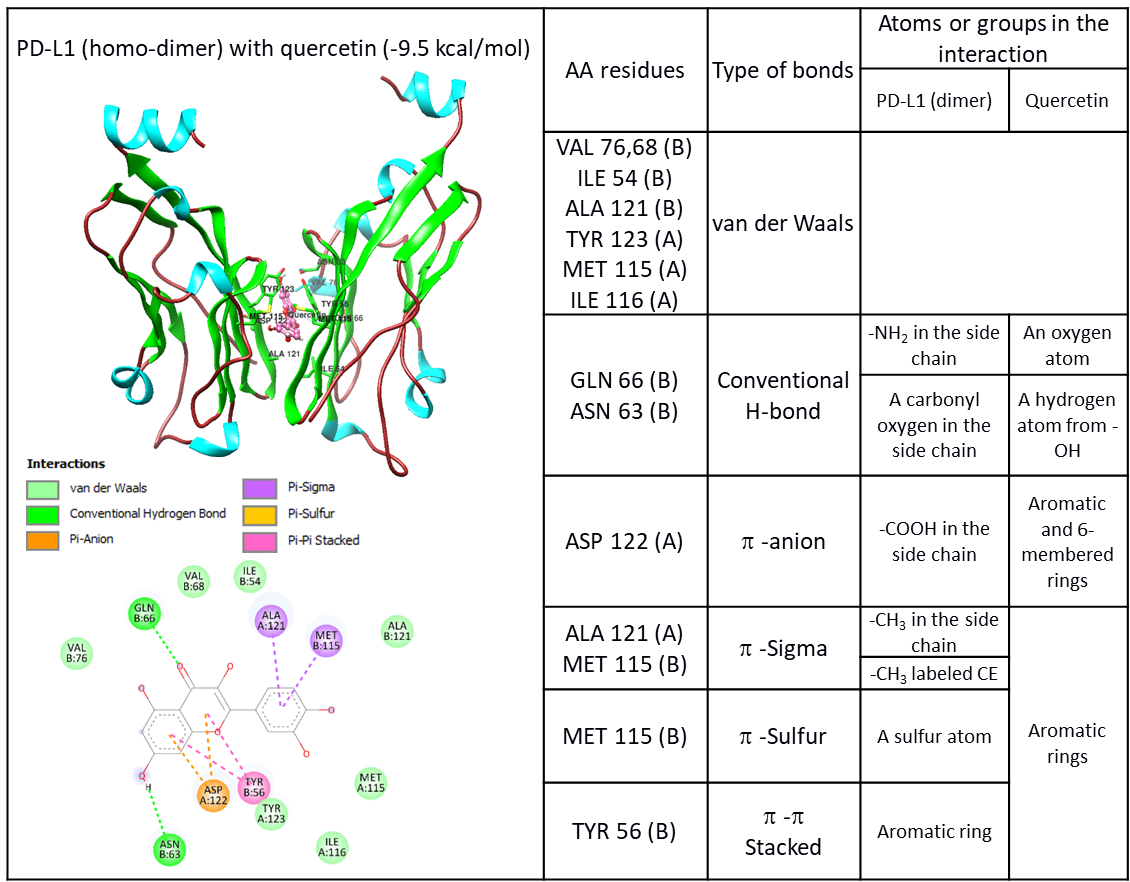
**

**Figure S11.** View of the docking pose of quercetin on the surface of the hPD-L1 symmetric homodimer (top left), schematic 2D diagram of the interactions with the receptor (bottom left). The right panel lists the interactions.


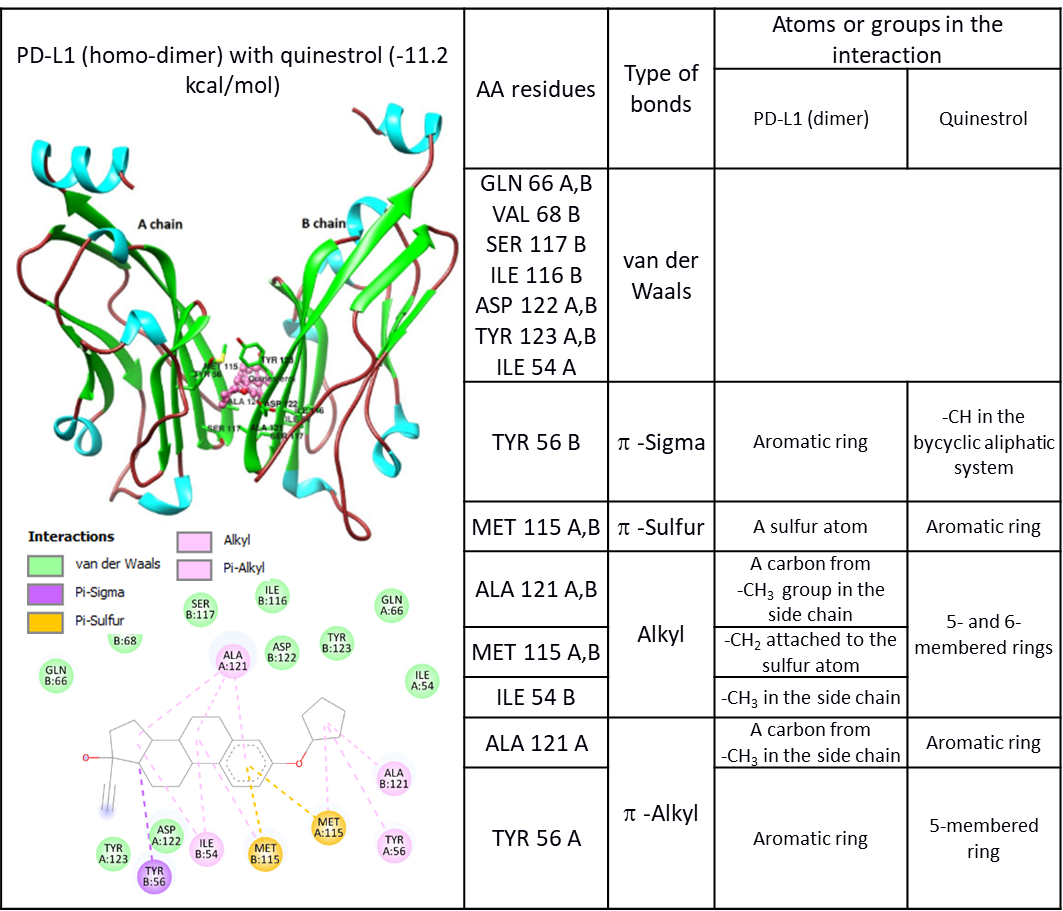


**Figure** **S12.** View of the docking pose of quinestrol on the surface of the hPD-L1 symmetric homodimer (top left), schematic 2D diagram of the interactions with the receptor (bottom left). The right panel lists the interactions.

**hPD-L1 N-terminal IgV fold domain**

**
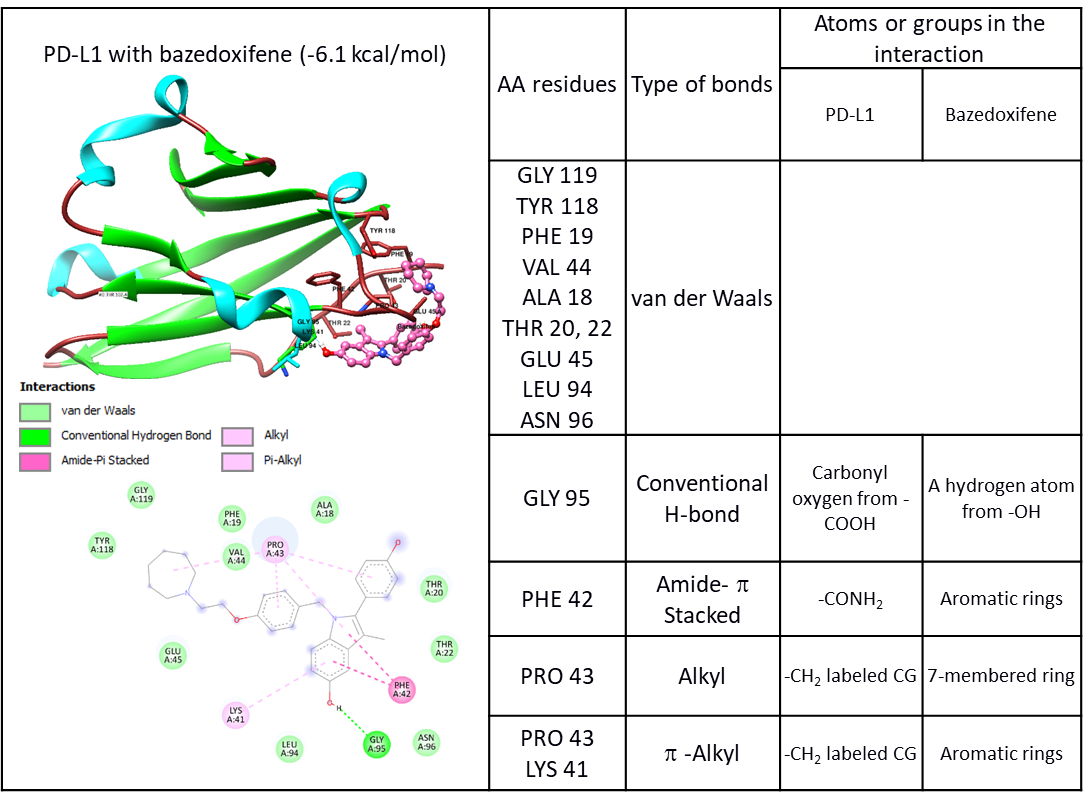
****Figure S13.** Docking pose of bazedoxifene on the surface of the hPD-L1 N-terminal IgV fold domain.

**
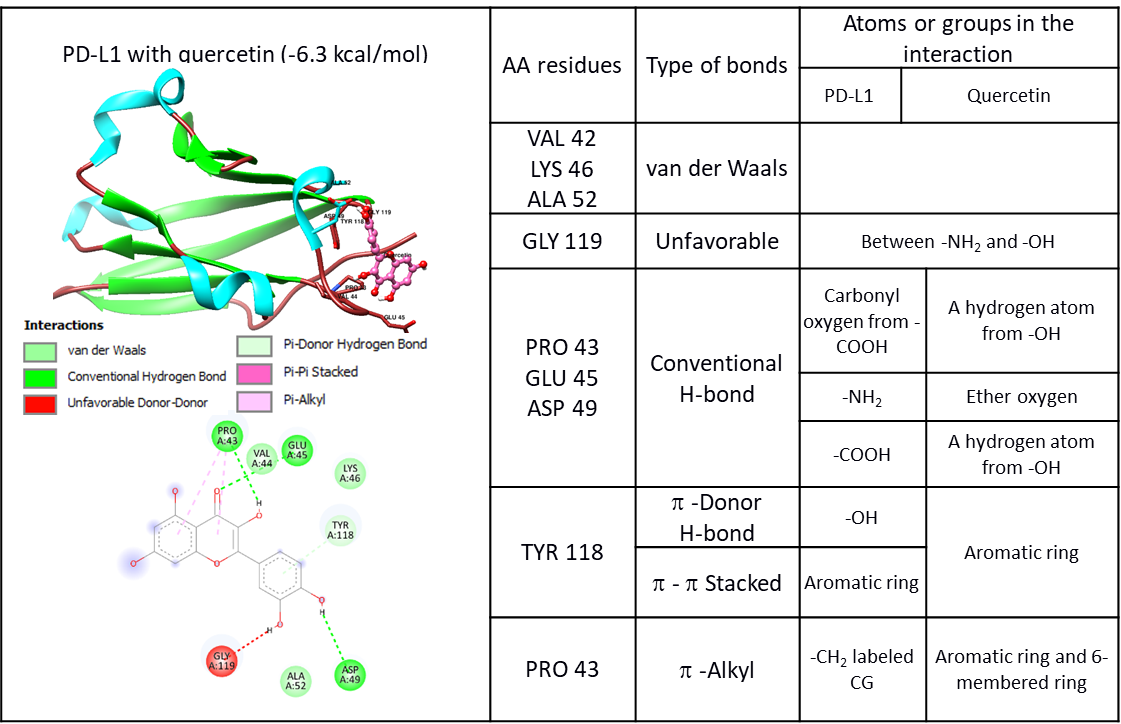
****Figure S14.** Scheme depicting the location of quercetin on the surface of the PD-L1 N-terminal IgV fold domain and a 2D diagram illustrating the interactions with the receptor.

**
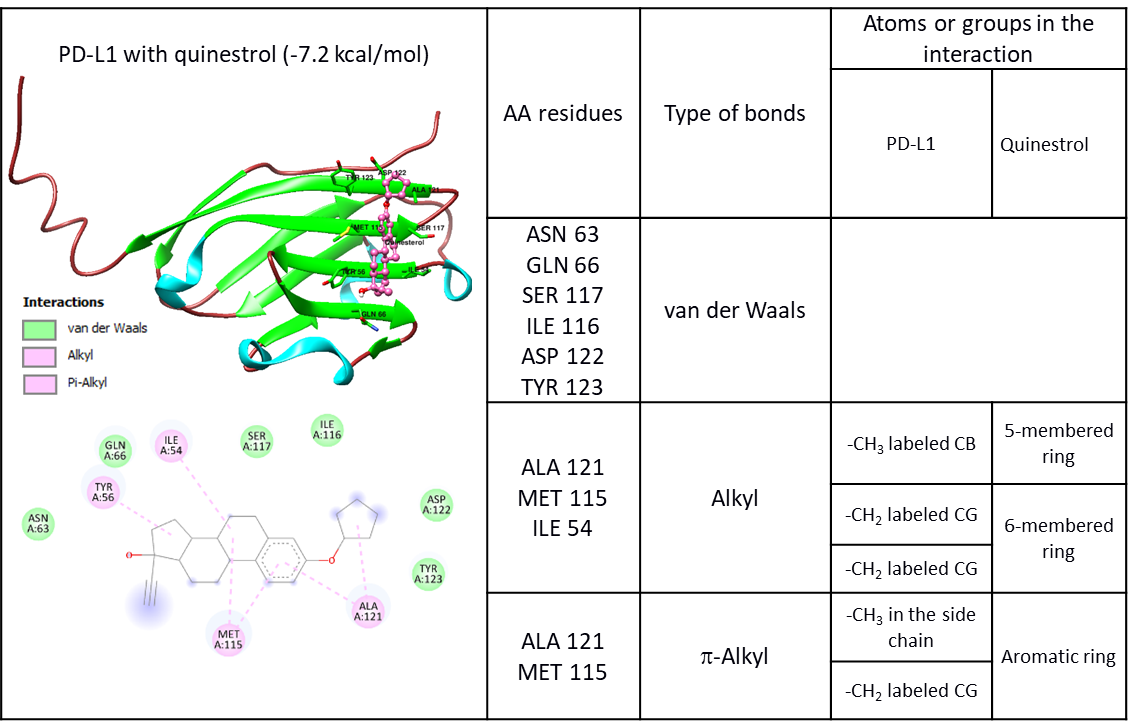
**

**Figure S15.** Scheme of the location of quinestrol on the surface of the PD-L1 N-terminal IgV fold domain and a 2D diagram illustrating the interactions with the receptor.

**PD-1**

**
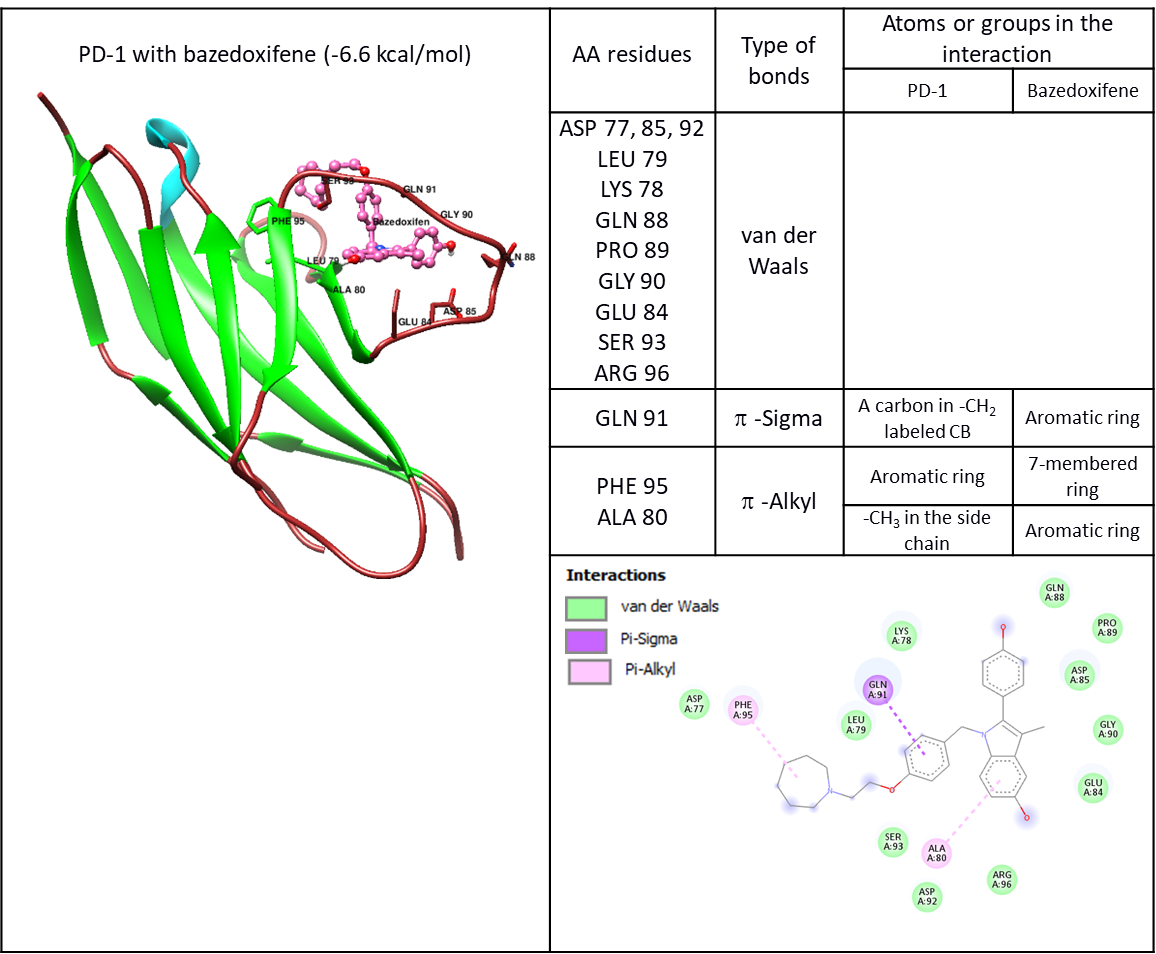
****Figure S16.** Scheme of the location of the bazedoxifene on the surface of the PD-1 and 2D diagram of the interactions with receptor.

**
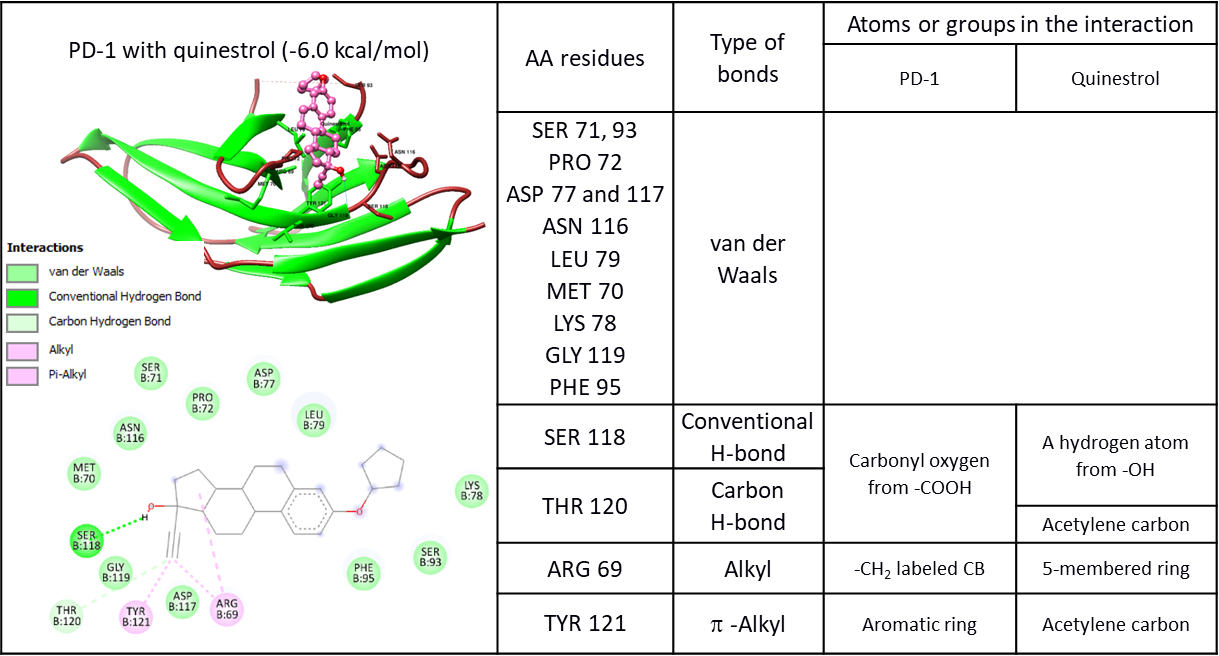
**

**Figure S17.** Scheme of the location of the quinestrol on the surface of the PD-1 and 2D diagram of the interactions with receptor.


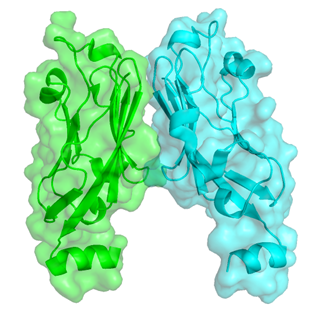


**Figure S18.** Representation of the PD-L1 symmetric homo-dimer with the central channel.

**References**

1. Jiao, P., et al., *Integrating Network Pharmacology and Experimental Validation to Elucidate the Mechanism of Yiqi Yangyin Decoction in Suppressing Non-Small-Cell Lung Cancer.* Biomed Res Int, 2023. **2023**: p. 4967544.

2. Liu, M., et al., *Investigation of the Underlying Mechanism of Huangqi-Dangshen for Myasthenia Gravis Treatment via Molecular Docking and Network Pharmacology.* Evid Based Complement Alternat Med, 2023. **2023**: p. 5301024.

3. Alnusaire, T.S., et al., *Revealing the Underlying Mechanism of Acacia Nilotica against Asthma from a Systematic Perspective: A Network Pharmacology and Molecular Docking Study.* Life (Basel), 2023. **13**(2).

4. Khalid, H.R., et al., *Integrated System Pharmacology Approaches to Elucidate Multi-Target Mechanism of Solanum surattense against Hepatocellular Carcinoma.* Molecules, 2022. **27**(19).

5. Tan, X., et al., *Mechanisms of Quercetin against atrial fibrillation explored by network pharmacology combined with molecular docking and experimental validation.* Sci Rep, 2022. **12**(1): p. 9777.

6. Pan, J., et al., *Qingfei Jiedu decoction inhibits PD-L1 expression in lung adenocarcinoma based on network pharmacology analysis, molecular docking and experimental verification.* Front Pharmacol, 2022. **13**: p. 897966.
